# Re-imagining fMRI for awake behaving infants

**DOI:** 10.1038/s41467-020-18286-y

**Published:** 2020-09-09

**Authors:** C. T. Ellis, L. J. Skalaban, T. S. Yates, V. R. Bejjanki, N. I. Córdova, N. B. Turk-Browne

**Affiliations:** 1 Department of Psychology, Yale University, New Haven, CT 06511 USA; 2 Department of Psychology, Hamilton College, Clinton, NY 13323 USA

**Keywords:** Cognitive neuroscience, Psychology

## Abstract

Thousands of functional magnetic resonance imaging (fMRI) studies have provided important insight into the human brain. However, only a handful of these studies tested infants while they were awake, because of the significant and unique methodological challenges involved. We report our efforts to address these challenges, with the goal of creating methods for awake infant fMRI that can reveal the inner workings of the developing, preverbal mind. We use these methods to collect and analyze two fMRI datasets obtained from infants during cognitive tasks, released publicly with this paper. In these datasets, we explore and evaluate data quantity and quality, task-evoked activity, and preprocessing decisions. We disseminate these methods by sharing two software packages that integrate infant-friendly cognitive tasks and eye-gaze monitoring with fMRI acquisition and analysis. These resources make fMRI a feasible and accessible technique for cognitive neuroscience in awake and behaving human infants.

## Abstract (web summary)

Using fMRI on awake infants could help us understand the contents of a baby’s mind, long before they can speak. Here, the authors report advances in how to collect, preprocess, and analyze task-based fMRI data from infants, and they share the resulting datasets and software.

## Introduction

Infants may hold the key to understanding the origins and functions of the human mind, and yet they are difficult to study because they cannot communicate verbally and have a limited repertoire of actions. Research on infant cognition has navigated these constraints by relying on simple behavioral measures, such as looking^1^ and reaching^2^. This approach has been supplemented by neural measures from electroencephalography^3^ and functional near-infrared spectroscopy^4^, which provide a window into infant cognition through the scalp without requiring external behavior. Functional magnetic resonance imaging (fMRI) stands to complement these scalp-based techniques, with sensitivity throughout the whole brain (including ventral surfaces and deep brain structures) and relatively high spatial resolution that can be linked to detailed anatomy. These advantages could dramatically improve our understanding of infant cognition^5,6^ — yet fMRI has rarely been used for this purpose.

There are many challenges to collecting fMRI data from infants: head and body motion, limited attention span, fussiness, inability to understand or follow instructions, high acoustic noise levels, and a lack of analysis approaches optimized for the infant brain. Several labs have avoided some of these constraints by performing fMRI experiments with infants or toddlers who are sedated^7^ or sleeping^8–10^. These studies have found innovative ways to investigate aspects of early cognitive development, such as by cuing previously learned episodes during sleep^9^. There are also now several large-scale projects that seek to understand functional brain development in infants during rest (HEALthy Brain and Child Development Study, UNC/UMN Baby Connectome project^11^ and Developmental Human Connectome Project). However, many more active forms of cognition, such as visual perception, selective attention, memory encoding, decision-making, and theory of mind, can only be manipulated and measured in awake infants. For this reason, we endeavored to create an approach that would make awake fMRI a feasible and accessible technique for cognitive neuroscience in infants.

The challenges above severely limit the amount of awake fMRI data that can be obtained from infants. Under the best of circumstances, it is not unusual for infants to fuss out after a few minutes of a task, compared to 60–90 min of fMRI data typically acquired from adults. In those precious few minutes, motion of the head or body, startle responses to noise, separation from parents, crying from hunger, teething or soiled diapers, and fatigue or napping, can reduce the amount of usable data to near zero. This assumes that data collection begins in the first place—some infants are not willing to lie down or wait through initial steps for the task to start. In sum, the rates of data exclusion and subject attrition create a risk that insufficient data will be collected in order to detect what are, even in adults, relatively small and noisy signatures of cognition in the brain.

The time is right to address these challenges because of the possibility of building on innovations in multiple domains. First, there have been significant advances in the preprocessing and analysis of adult fMRI data. For example, beyond localizing functions in the brain, multivariate methods from machine learning have made it possible to extract and interpret the mental contents of neural activity patterns (e.g., specific percepts^12^, memories^13^, and decisions^14^). Second, substantial methodological and theoretical groundwork has already been done in developmental psychology. This includes infant-friendly behavioral tasks whose underlying neural foundations are mostly unknown, but for which such data could help resolve ongoing debates, such as about novelty vs. familiarity preferences in looking time^1,15^. Third, the equipment and software platforms exist to create custom, open-source solutions for data acquisition, stimulus presentation, and behavioral monitoring. This allows the scanning environment and experimental protocols to be redesigned for infants from the ground up.

Some of our procedures were inspired by previous infant fMRI studies, others by studies in populations with related constraints (e.g., patients and animals), and yet others are unique and the result of trial and error. No one aspect of our set up is sufficient in our experience, so here we report a family of methods that collectively allow for the robust collection of fMRI data from awake, behaving infants. Together, these procedures ensure sufficient flexibility to adapt task selection, duration, and breaks to the infant’s temperament and attention span, while also being rigorously consistent across participants in the environment, apparatus, personnel, within-task design, stimuli, MRI acquisition parameters, and data preprocessing and analysis. In addition to describing these methods in detail, all of the code needed to implement them has been made publicly available. This includes an experiment menu system that flexibly incorporates infant-friendly cognitive tasks and seamlessly coordinates stimulus presentation, behavioral monitoring, and scanner synchronization, as well as a semi-automated pipeline tailored for the analysis of the resulting infant data. We have deployed these methods at three scanning sites, with findings from the first two completed cohorts reported here. These two fMRI datasets from awake infants have also been made publicly available. Our hope is that these software and data resources, combined with included recommendations about recruitment, safety, equipment, task design, personnel, preprocessing, and more, will help make fMRI a more prevalent technique for studying the early developing mind.

## Results

### Overview

Below we describe two components of our work in awake, infant fMRI: First is the protocol itself, including apparatus, procedures, and algorithms used for data acquisition and analysis. We describe the protocol in full detail in the Methods section, but discuss aspects here when they could themselves be considered a result or when needed to understand the presented data. Second is our initial results, including quality assurance tests and statistical analyses used to evaluate our approach. We report four metrics: (i) the quantity of infant data, and quality relative to adult data; (ii) the reliability of BOLD activity evoked in visual tasks; (iii) how these visual responses vary across preprocessing decisions in one dataset; and (iv) a replication in a second dataset from an overlapping age range and new site. Our hope is that the methods described herein, and the code to implement them that we are releasing with this paper, will accelerate the adoption and refinement of awake infant fMRI.

### Data quantity and quality

We report data from two cohorts of infants acquired sequentially from different sites. Cohort I was collected on a 3T Siemens Skyra MRI at Princeton University over a broad age range (6–36 months). Cohort II was collected on a 3T Siemens Prisma MRI at Yale University from a younger and narrower age range (3–12 months). Given the small number of studies of this type, we treated Cohort I as an exploratory sample in which to search for good analysis parameters and then applied these parameters in a principled way to Cohort II. The data were analyzed with a custom software pipeline (Supplementary Fig. 1), using code that we are releasing with this paper.

We collected two types of scans in each session (Supplementary Table 1): an anatomical image using a T1-weighted pointwise encoding time reduction with radial acquisition (PETRA) sequence^16^, and functional data using an echo planar imaging (EPI) sequence while infants performed cognitive tasks. The PETRA sequence (part of the Siemens Quiet Suite) was more robust to motion than other anatomical sequences (e.g., MPRAGE) in early piloting, we suspect because it is short and samples K-space radially (Supplementary Fig. 2); that said, any T1- or T2-weighted anatomical sequence can be used in the analysis pipeline. The EPI sequence was standard, although the provided experiment and analysis code is compatible with a range of parameters (only TR duration needs to be specified), allowing for higher resolution, faster sampling, and/or greater acceleration.

**Fig. 1:**
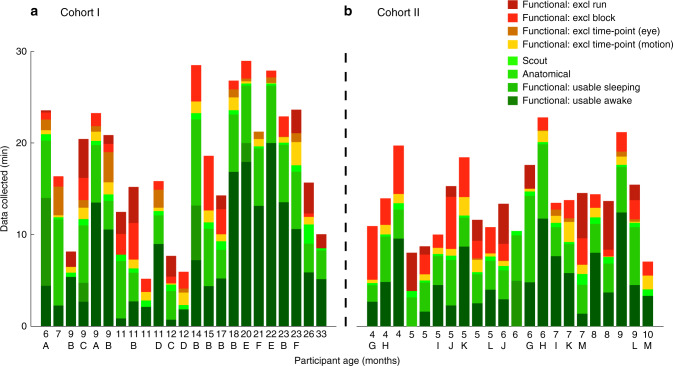
Cumulative data retention for individual scanning sessions. For **a** Cohort I and **b** Cohort II, the age of the child in months is shown on the *x* axis and the duration of the scan on the *y* axis, including a breakdown by color for different categories of data. Functional time-points were deemed usable when the translational motion was below 3 mm (the voxel resolution) and when the infant’s eyes were open and on-screen (determined by manual gaze coding of a video recording of the infant’s face). Epochs of data during a task (blocks in a block design or trials in an event-related design) were excluded if more than 50% of the time-points were excluded because of motion and/or eye-gaze. Runs were excluded if no blocks were usable within a run. If the infant fell asleep during functional scans, we occasionally continued collection and labeled it as resting data. Anatomical and scout scans are included if they were completed, although not distinguished based on quality. Individual infants who completed more than one session have been assigned a letter code, which is shown beneath the age of each of their sessions (no letter means the infant from that session only participated once).

In Cohort I (Fig. 1a; Supplementary Table 2), we scanned 11 distinct individuals between 6 and 33 months (*M* = 15.2 [*SD* = 6.9]) across 23 sessions (1–8 sessions per participant). Five additional sessions could not be used because the child did not go in the scanner. Of the participants who were scanned, an average of 31.3% (13.0 min) of the total time when experiment code was running (proxy for how long we were trying to scan) produced usable functional and/or anatomical data. On average, 7.7 mins of awake functional data were usable; this duration is in the ballpark of typical infant cognition studies with behavioral or other neuroimaging techniques^17–19^. After preprocessing, 16 of the 28 sessions resulted in the acquisition of at least one full experiment’s worth of usable data. Because some participants completed more than one experiment per session (range: 0–3), our overall retention rate of usable experiments per scheduled session was 1.18. Although the amount of data in minutes per experiment is low relative to adult fMRI, we obtained slightly more than one useful infant experiment dataset per session on average.

Beyond obtaining a reasonable quantity of data in Cohort I, we also explored whether the data were of sufficient quality. One unique aspect of our apparatus is that we did not use the top of the head coil, as typical with adults. This reduces the number of coil elements, which in principle reduces the amount of signal received from brain regions that would have been nearby. This was a deliberate decision made to increase infant comfort, provide parents an unobstructed view of the infant’s face, and enable ceiling-based visual stimulation and reliable camera-based eye-tracking. Signal loss would only be expected in the anterior portion of the brain most distant from the remaining bottom coil elements. Such loss may be partly mitigated by the small size of the infant brain, which reduces this distance. In fact, if the top coil had been used, it would have been far from the infant’s forehead, minimizing the impact of these elements on image acquisition.

**Fig. 2:**
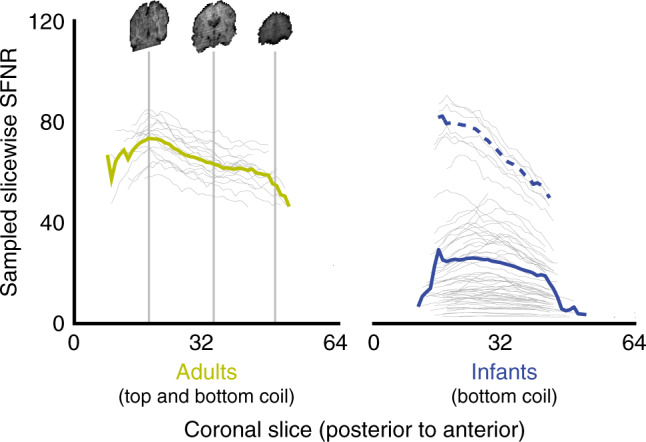
Comparison of signal-to-fluctuation-noise ratio (SFNR) in adults and infants. Adults had both the top and bottom coils attached, while infants only had the bottom coil. SFNR was computed for each voxel and then the values from a random sample of 1000 voxels in each coronal slice of at least that size were averaged, spanning the posterior-anterior axis of the brain. Each gray line is one run from one participant (*N* = 64 for infants, *N* = 16 for adults) and the colored lines represent the average. The solid blue line is the mean of all infant runs, whereas the dashed blue line is the mean of five low-motion runs.

Given this non-traditional approach, we evaluated signal quality from the bottom coil. A common metric for describing the quality of fMRI data is the signal-to-fluctuation-noise ratio (SFNR)^20^: the magnitude of the BOLD signal in a brain voxel relative to its variability over time. We compared voxelwise SFNR in our infant data (*N* = 64 runs) with only the bottom coil connected against a gold standard of adult data (*N* = 16 runs) with both bottom and top coils. The adult data were obtained from a published study^21^ using the same scanner and EPI sequence as the infants in Cohort I. We compared overall SFNR across groups and examined how SFNR changed along the posterior-anterior axis of the brain, which tracks increased distance from the bottom coil (Fig. 2).

SFNR was higher in adults (*M* = 66.0) than infants (*M* = 23.6) over the whole brain (*F*(1,77) = 76.28, *p* < 0.001). This likely reflects factors beyond just the coil, such as age-related differences in head motion and BOLD contrast. Head motion is especially problematic because it can introduce dramatic variability, as a result of signal loss from motion within a volume acquisition and by changing the anatomical location of voxels in the field-of-view (FOV), including across tissue boundaries. Indeed, in a subsample of 5 runs that had at least 50 TRs and less than 0.2 mm of translational motion across the run on average (dashed line in Fig. 2), SFNR was similar to adults with the top of the head coil attached (*M* = 68.5). Indeed, there is a strong negative rank correlation between average translational motion and average SFNR (Spearman’s *ρ* = −0.97, *p* < 0.001). This suggests that removing the top coil did not guarantee lower signal quality, and that taking steps to mitigate motion is most important to increasing signal quality. Moreover, despite being lower overall, infant SFNR was mostly in a usable range and not dramatically different from the early days of adult fMRI^20^.

SFNR was higher in the posterior (*M* = 34.3) than anterior (*M* = 30.1) half of the brain (*F*(1,77) = 47.46, *p* < 0.001). If caused by the lack of a top coil, we would expect a larger posterior-to-anterior drop in infants than adults with both bottom and top coils. However, the drop was if anything smaller in infants than adults in both proportional (adult *M* = 0.88, infant *M* = 0.92, Welch’s *t*(35.0) = 1.96, *p* = 0.053) and absolute terms (*F*(1,77) = 11.84, *p* < 0.001). Different from infants, when adult data are collected without the top of the head coil attached, the drop-off is severe (Supplementary Fig. 3). We obtained consistent results with signal-to-noise ratio (SNR) in place of SFNR (Supplementary Fig. 4). Thus, we found no additional hit to anterior sensitivity when using only the bottom coil in infants.

### Visual activity

We further evaluated data quality by examining basic neural responses. The tasks we tested in these cohorts all involved visual stimuli and so we expected to observe responses in visual cortex, including early visual cortex (V1) and lateral occipital cortex (LOC). To quantify responses in these regions of interest (ROIs), we estimated the BOLD activity evoked by task epochs using a general linear model (GLM). To assess the selectivity of visual responses in the brain, we included a control ROI in early auditory cortex (A1), as all stimuli were silent.

In Cohort I, there were 14 sessions from 7 unique participants with one or more runs (32 total runs) that contained at least two usable task blocks (*M* = 4.5 blocks; range: 2–12). This corresponds to 72.4% of the functional data retained after motion, eye-tracking, and other exclusions during preprocessing. The preprocessed data for each run were trimmed to include blocks of visual stimulation (lasting 24–80s) separated by baseline rest (6s). Prolonged periods of movie viewing or sleep were not included in this analysis of evoked responses. Blocks of included data were modeled with a canonical hemodynamic response function based on prior infant neuroimaging studies^8,22,23^. ROIs were transformed into subject space to measure effects within individual participants. To examine group effects across the brain, functional data were aligned to age-specific infant MNI templates, which were, in turn, registered to adult standard MNI space to facilitate comparison across infant ages.

**Fig. 3:**
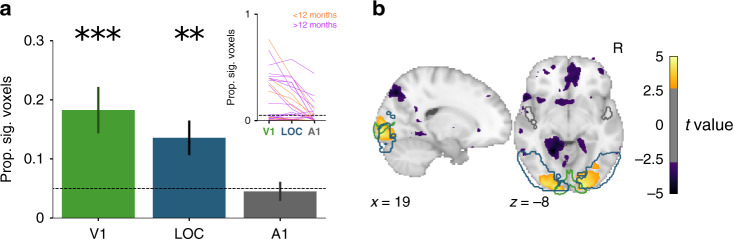
Visual evoked activity for each run from Cohort I. **a** Mean proportion of voxels showing significant visual responses within run (thresholded at *p* < 0.05) for V1, LOC, and A1 regions of interest (ROIs). Data are presented as mean values ± between-run standard error of the mean (SEM). Two-tailed bootstrap resampling compared against the chance proportion level (0.05): **p < 0.01, ***p < 0.001 for the *N* = 32 runs. Inset: change in proportion of significant voxels across the ROIs for each run for infants younger (orange) or older (magenta) than a year old. **b** *t*-value for voxels across the whole brain showing reliable responses across runs (two-tailed *p* < 0.005, uncorrected). V1, LOC, and A1 ROIs are outlined in green, blue, and gray, respectively.

For ROI analyses, we quantified the proportion of voxels in each region that showed a significant visual response within individual runs (Fig. 3a; Supplementary Table 3). These proportions were reliably different from chance (0.05) across runs in V1 (*M* = 0.18 [*SD* = 0.22], *p* < 0.001) and LOC (*M* = 0.14 [*SD* = 0.17], *p* = 0.001), but not A1 (*M* = 0.05 [*SD* = 0.09], *p* = 0.704). Compared to A1, the proportions were greater in V1 (25/32 runs, *p* < 0.001) and LOC (27/32 runs, *p* < 0.001); V1 was reliably greater than LOC (22/32 runs, *p* = 0.001). Whole-brain voxelwise analyses examining reliability across runs confirmed these findings and showed that effects were strongest in earlier visual regions (Fig. 3b). We also ran these analyses at the level of sessions rather than runs and found consistent, if not stronger, results (Supplementary Fig. 5a, b).

### Preprocessing parameters

There is an unavoidable trade-off in infant fMRI between the amount of data retained and the quality of the data included (see Supplementary Movie 1 for examples of different types of motion). We chose some parameters to be more liberal than typical adult fMRI (e.g., tolerating motion up to 3 mm), knowing that the inclusion of noisy data reduced the power of our analyses. Our reasoning was that infant data are so precious and difficult to obtain that it was worth giving rigorous statistical analyses the opportunity to find signal in the noise. Indeed, the results described in the preceding sections provide initial evidence that our pipeline can recover signal. Nevertheless, we wanted to explore the impact of our preprocessing decisions more fully. We compared the proportion of significant voxels in ROIs across parameter settings. A higher proportion was not our only consideration: more stringent criteria may improve signal and yet be unacceptable if they exclude too much data. Indeed, longitudinal designs are critical to infant research but would be severely hampered by missing data-points.

**Fig. 4:**
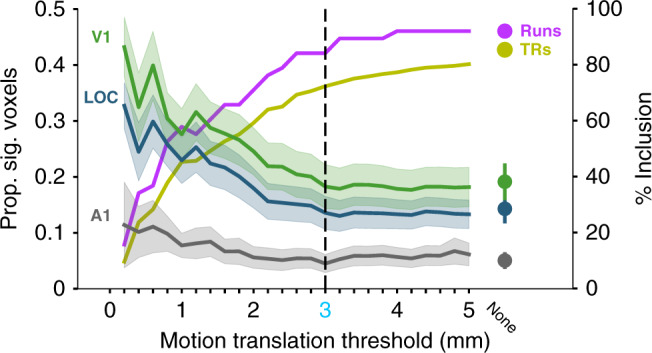
Proportion of significant voxels across translational motion thresholds. Values are separately shown for V1 (green), LOC (blue), and A1 (gray). The dashed line indicates the motion threshold that was used as the default. None indicates the results when no motion threshold was applied. The lefthand axis reports the proportion of significant voxels (*p* < 0.05) for each ROI. The righthand *y*-axis reports the proportion of included TRs in mustard and included runs in purple (out of 38 runs with at least 2 blocks). Note that regardless of the motion threshold, some blocks/runs were excluded because the infant’s eyes were closed. Data are presented as mean values ± between-run SEM as shaded area.

The proportion of voxels with significant visual responses varied with the threshold for motion exclusion (Fig. 4). Stricter thresholds increased the proportion of significant voxels in V1 and LOC. However, the amount of data excluded was severe: 0.2 mm had the highest proportion of significant voxels in V1, but only 7 runs (of 38) across 3 sessions (of 17) were retained with at least two task blocks (vs. 32 runs and 14 sessions for 3 mm). This reduced sample size may increase the susceptibility to noise, with a higher proportion of voxels in A1 showing significant visual responses. The same pattern of results was observed across sessions (Supplementary Fig. 6).

**Fig. 5:**
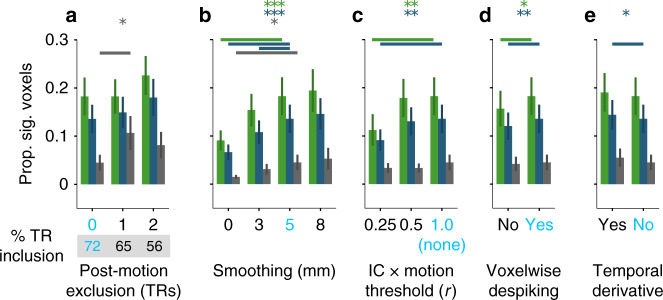
Proportion of significant voxels after various preprocessing decisions. Results are shown for V1 (green), LOC (blue), and A1 (gray). The parameter setting we used for the other analyses is shown in bright blue. **a** Number of time-points into the future removed following above-threshold motion, with the percentage of retained time-points below (*N* = 32, 30, and 25 for 0, 1, and 2 time-points removed, respectively). **b** Full-width half maximum (FWHM) of the Gaussian spatial smoothing kernel (*N* = 32). **c** Minimum correlation threshold for excluding independent components based on their relationship to motion parameters with lower values leading to more components excluded (*N* = 32). **d** Turning on or off AFNI’s voxelwise despiking (*N* = 32). **e** Inclusion of temporal derivatives in the design matrix of the general linear model (*N*= 32). Significance of one-tailed Chi-square test for omnibus linear mixed model: *p < 0.05, **p < 0.01, ***p < 0.001. Significant two-tailed simple effects between our chosen parameter setting (in bright blue) and other settings are indicated by a bold line (*p* <0.05). Data are presented as mean values ± between-run SEM.

We also evaluated how five other preprocessing parameters affect visual evoked activity using linear mixed effects models. First, during motion exclusion there was no benefit of excluding additional time-points up to 4s (2 TRs) after the time-point with above-threshold motion (Fig. 5a) in V1 (*χ*^2^(2) = 0.67, *p* = 0.714) or LOC (*χ*^2^(2) = 0.97, *p* = 0.617), though there was in A1 (*χ*^2^(2) = 7.33, *p* = 0.026; 0 vs. 1: *t*(56.01) = 2.57, *p* = 0.013); post-motion exclusion further reduced the number of time-points retained. Second, spatial smoothing (Fig. 5b) increased the proportion of significant voxels in V1 (*χ*^2^(3) = 41.16, *p* < 0.001), LOC (*χ*^2^(3) = 51.27, *p* < 0.001), and A1 (*χ*^2^(3) = 11.06, *p* = 0.011), with our chosen default (5 mm) reliably better than no smoothing (0 mm) in V1 (*t*(96.00) = −5.20, *p* < 0.001), LOC (*t*(96.00) = −5.70, *p* < 0.001) and A1 (*t*(96.00) = −2.45, *p* = 0.016) and better than less smoothing (3 mm) in LOC (*t*(96.00) = −2.29, *p* = 0.024). Third, denoising with independent components related to motion (Fig. 5c) hurt V1 (*χ*^2^(2) = 12.73, *p* = 0.002; 0.25 vs. 1.0: *t*(64.00) = −3.17, *p* = 0.002) and LOC (*χ*^2^(2) = 7.51, *p* = 0.023; 0.25 vs. 1.0: *t*(64.00) = −2.51, *p* = 0.015) but not A1 (*χ*^2^(2) = 1.23, *p* = 0.540). Fourth, voxelwise despiking (Fig. 5d) helped V1 (*χ*^2^(1) = 6.12, *p* = 0.013) and LOC (*χ*^2^(1) = 8.39, *p* = 0.004), but not A1 (*χ*^2^(1) = 0.68, *p* = 0.408). Fifth, including temporal derivatives in the GLM (Fig. 5e) had a marginal effect in V1 (*χ*^2^(1) = 3.60, *p* = 0.058), a significant effect in LOC (*χ*^2^(1) = 6.04, *p* = 0.014), and no effect in A1 (*χ*^2^(1) = 0.91, *p* = 0.341). We repeated these analyses across sessions rather than runs (Supplementary Fig. 7). Together, these findings indicate that the parameters we chose for our pipeline were sensible, at least with respect to univariate analyses of visual cortex.

### Generalization

Cohort I was collected first and was used to determine preprocessing parameters that balanced data quality and quantity. We then applied these parameters in Cohort II (Fig. 1b; Supplementary Table 2), to test whether they enabled analogous success in new participants, generalizing to a consistently younger population and a new site. Cohort II also contained more sessions in which multiple experiments were performed during the same functional run. These runs were trimmed into the time-points corresponding to each experiment and preceding/subsequent rest, leading to experiment-specific *pseudo-runs* that were submitted for preprocessing. We scanned 15 new individuals between 4 and 10 months old (*M* = 6.05 [*SD* = 1.8]) across 22 sessions (1–2 sessions per participant). In these sessions, 22.0% of the total time (9.2 min) produced usable functional and/or anatomical data on average. On average, 4.9 mins of awake functional data were usable. There was one additional session where the participant did not go into the scanner (resulting in a total of 23 sessions). Of these, 14 sessions resulted in the acquisition of at least one full experiment’s worth of usable data. A variable number of experiments were collected per session (range: 0–3), resulting in a retention rate of 1.04 useful experiments per scheduled session on average.

**Fig. 6:**
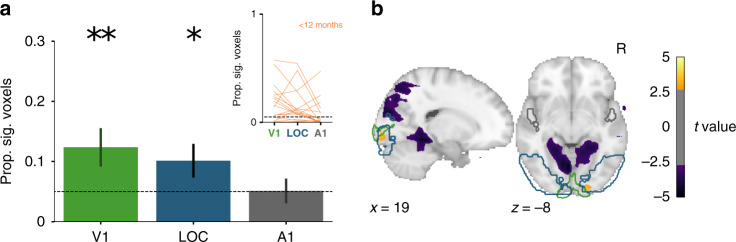
Visual evoked activity for each run from Cohort II (akin to Fig. 3 for Cohort I). **a** Proportion of voxels showing significant visual responses within run (thresholded at *p* < 0.05) for V1, LOC, and A1. Data are presented as mean values ± between-session SEM. Two-tailed bootstrap resampling compared against the chance proportion level (0.05): *=*p* < 0.05, **=*p* < 0.01 for *N* = 26 runs. Inset: change in proportion of significant voxels across the ROIs for each run (all infants younger than a year old). **b** *t*-value for voxels across the whole brain showing reliable responses across runs (two-tailed *p* < 0.005, uncorrected). V1, LOC, and A1 ROIs are outlined in green, blue, and gray, respectively.

There were 18 sessions from 13 unique participants with one or more runs (total of 26 runs) that contained at least two task blocks (*M* = 4.1 blocks per run; range: 2–12) that could be used to analyze visual evoked activity. The amount of usable data corresponds to 64.6% of the functional data retained after preprocessing with the parameters chosen in Cohort I (bright blue in Fig. 5). We again quantified the proportion of voxels in each bilateral ROI that showed a significant visual response within run (Fig. 6a and Supplementary Table 4). Replicating Cohort I, these proportions reliably differed from chance (0.05) across runs in V1 (*M* = 0.12 [*SD* = 0.16], *p* = 0.007) and LOC (*M* = 0.10 [*SD* = 0.14], *p* = 0.038), but not A1 (*M* = 0.05 [*SD* = 0.11], *p* = 0.970). Compared to A1, the proportions were greater in V1 (13/26 runs, *p* = 0.007) and LOC (21/26 runs, *p* = 0.037); V1 did not differ from LOC (9/26 runs, *p* = 0.307). Voxelwise analyses again revealed reliable neural responses, predominantly in right visual cortex (Fig. 6b). Similar ROI and voxelwise results were obtained across entire sessions rather than runs (Supplementary Fig. 5c, d). Thus, we were able to retain a substantial amount of data and recover task-evoked visual responses in young infants with pre-planned acquisition and analysis methods.

## Discussion

We presented how to acquire and analyze infant fMRI data, with evidence from two cohorts that these methods can produce high retention rates and reliable neural responses across different infant ages. To address the unique challenges of this population, we adapted the scanning environment and our analysis approach in several ways. Despite deviating from standard protocols and requiring custom equipment and software, these adaptations can be implemented at reasonable cost in most scanning facilities. The main costs include the bore ceiling projector setup, the eye-tracking camera video feed, and the vacuum pillow/pump system. Existing projectors and eye-trackers can be re-purposed by adjusting the mounting, calibration, and/or lenses. Indeed, we have implemented these methods successfully at three sites that differed in size and configuration, and we are starting to work with colleagues to implement them at other institutions.

If adopted by the research community, awake infant fMRI could shed new light on central, long-standing questions about cognitive development by providing more direct access to infants’ internal mental representations. This could enable progress in understanding how infants perceive^24^ and categorize^25^ the world, make predictions^26^ and run mental simulations^27^, and infer the mental states of others^28^. Better understanding the infant mind and brain could in turn shed light on cognitive neuroscience more generally^5^, for example, by informing theories of memory about the early functioning of brain systems, such as the hippocampus that might help explain later infantile amnesia^29^. To facilitate uptake, we have released our code for data acquisition, a flexible experiment menu system. We have also released our code for data analysis, a semi-automatic pipeline that handles the noise characteristics and unpredictable nature of infant fMRI. These software packages are a key contribution of this work, along with other innovations, both technical (e.g., immersive visual display with eye-tracking) and non-technical (e.g., procedures to enhance infant and parent comfort). Although not their original purpose, these tools can also be used in adults for both behavioral and fMRI experiments, including in patient populations. As such, they represent a general-purpose infrastructure for experimental design and analysis both within and outside the field of infant fMRI.

These advances allowed us to acquire considerable fMRI data from awake, behaving infants. We have shared these data, the first substantial public datasets of this type. The two cohorts reflect three years of data collection effort, from 26 unique infants who participated in 45 sessions and completed 57 experiments. The results of individual experiments await further data collection and specialized analysis, and will be reported in forthcoming papers. However, the initial analyses reported here as a proof-of-concept (collapsing across experiment details) reveal reliable and selective univariate visual responses in human infants as young as four months.

Whether our approach is appropriate for other types of analysis (e.g., multivariate), tasks (e.g., auditory), and brain regions (e.g., prefrontal cortex) remains an open question. For example, other groups working with infant fMRI data have used stricter thresholds for motion exclusion^22,30^. Similarly, large motion transients can be excluded at the time-point level, as done here, or used to cleave runs into pseudo-runs with low motion^30^. Moreover, our use of only the bottom half of the head coil, although effective for investigating visual activity, may not be optimal for all task designs, such as when focused on frontal activity in older infants and children with larger brains. Custom head coils are one compelling solution^30^. We hope that the proposed methods, including shared code and data, will increase the feasibility and prevalence of infant fMRI, engaging more researchers to help converge on standards and best practices. At the same time, we recognize that some important developmental topics, such as motor learning and caregiver interactions, are better investigated with other procedures and neuroimaging technologies.

In the main analyses reporting visual evoked activity, we presented results of analyses computed on each run independently. Requiring reliable responses within individual runs is a conservative test of data quality, as adult data are typically aggregated across multiple runs and participants. We conducted the analysis this way to emulate short experiments consisting of a few blocks within a run. However, we also observed reliable responses over longer periods of data collection, across entire sessions. Most of our infant fMRI experiments can be completed within session across one or more runs, hence run-wise and session-wise analyses represent bounds on the amount and structure of the data per participant. Despite the fact that these analyses collapsed across a variety of visual stimuli and participant ages, the findings indicate that our protocol can produce robust fMRI activity in infant visual cortex.

Although these methods proved successful for collecting functional data from awake infants, a limitation of our approach is that anatomical images are generally lower quality than from adults or sleeping infants. Given the limited attention span of infants, we prioritized speed over quality when setting up our anatomical sequence. Longer scans that produce higher-quality images in a participant who remains still would, in our experience, result in worse images in infants, compared to shorter scans, because of an increasing likelihood of motion as the scan progressed. The anatomical images we collected were almost always sufficient for aligning functional data, but there is a need for better short anatomical sequences to enable the reconstruction of cortical surfaces and segmentation of subcortical structures. A different solution could be to complete portions of the session with the top half of the head coil attached. For example, the functional scans could be performed first when the infant arrives awake, with the anatomical scan saved until later in the session in case the infant falls asleep (or sleep is induced). Nevertheless, the anatomical scans included in the datasets we have shared may allow others to improve registration algorithms for images with motion noise. Indeed, this release of infant anatomical scans itself represents a significant increase in the amount of such data publicly available.

We think that one of the most important features of our protocol is the flexibility with which it can adapt to the unpredictable temperament of infants. However, this comes at the cost of increased variability between sessions. Any given experiment is often preceded by different events across participants, such as whichever cartoon they happened to like most during scanner calibration. This variability is unfortunate but likely unavoidable, as it is essential in our experience both to entertain infants during the downtime between scans and to change tasks when they do not like or get bored with stimuli. A related source of variability comes from the fact that we aim to collect multiple experiments per session and yet it is hard to know in advance which tasks will be tolerated and when. Without the ability to enforce counterbalancing of task order, it is possible that the experiments could interact in some way that biases results or adds noise. We do take steps to mitigate this concern, such as pseudo-randomizing the planned experiment sequence across participants and using different classes of visual stimuli across experiments to reduce habituation, but we recognize that this partial solution may be undesirable for some studies.

In sum, the methods described in this paper, along with the code and data released as companion resources, should make it easier for groups interested in infant fMRI to enter this field. Given our limited understanding of the infant brain, and the success of fMRI in adults, infant fMRI has the potential to provide revolutionary insights into the origins and nature of the human mind.

## Methods

### Participants

New data were collected in two cohorts of infants: Cohort I from the Scully Center for the Neuroscience of Mind and Behavior at Princeton University and Cohort II from the Magnetic Resonance Research Center at Yale University. In Cohort I, 11 unique infants (5 females) aged 6 to 33 months were scanned across 23 sessions (1–8 sessions per participant). Not included in this total were five sessions without fMRI data because the infant would not lie down (4 additional unique infants, 1 infant included above who contributed a usable session on another occasion). In Cohort II, 15 unique infants (8 females) aged 4 to 10 months were scanned across 22 sessions (1–2 sessions per participant). One other session was excluded because the infant would not lie down (1 additional unique infant). The parent(s) and/or guardian(s) of each infant in a cohort provided informed consent to a protocol approved by the Institutional Review Board of each university. Previously published data^21^ collected from 16 adults at Princeton University were re-analyzed for SFNR comparison.

### Orientation session

When a family expressed interest in participating, we first brought them in for an orientation session. This involved meeting a member of our team to discuss research goals, review procedures and safety measures, answer questions, and complete forms (informed consent and preliminary metal screening).

We typically then introduced the family to the scanning environment using a mock scanner, which consisted of a plastic shell that looked like a scanner but lacked the magnet and other hardware (Psychology Software Tools). This system also allowed for playback of scanner sounds to let parents know what the scanner sounded like. A parent placed the infant on their back on a motorized scanner table, which we then slid into the simulated bore. This helped us judge the infant’s ability to lie still. This also helped us judge the parent’s comfort level with separation, though the infant always remained within arm’s reach. We have now shifted away from the mock scanner to a simpler, less cumbersome system that has proven equally effective in infants. Specifically, we created a simulated bore from a 55-gallon white plastic barrel that was sawed in half lengthwise (into a half circle tunnel), into which we inserted a clear plastic window to show a screen. The parent places the infant on a changing mat inside the tunnel.

We did not use the infant or parent response to the mock scanner/tunnel for formal screening purposes. Although we monitored infant behavior and parental comfort throughout, these casual observations were not predictive of future scanning success. Mock scanning can be helpful in children older than two years^31,32^, although not always^33^. Indeed, our experience with infants has been that success is primarily determined by factors that are variable from session to session. This is likely because of dramatic developmental changes every few weeks and because of idiosyncratic factors related to sleep, hunger, illness, teething, time of day, etc.

### Hearing protection

The final part of the orientation was to familiarize the infant with hearing protection, which parents were encouraged to continue practicing at home. Our goal was to reduce the sound level they experienced during scanning to the range of daily experiences (e.g., musical toys, daycare environments, walking on the street). Sounds around 70 dB are thought to be safe, whereas sounds at or above 85 dB (roughly a loud school environment^34^) could cause damage after extended exposure without protection^35^. Although it is theoretically possible for MRI machines to exceed 110 dB (roughly a loud sports stadium^36^), sound insulation and sequence selection can result in lower levels. Indeed, in our scanning environments and for the sequences we used, the measured sound pressure level reached a maximum of 90 dB.

With hearing protection, it is possible to reduce the sound level by 33 dB, bringing even the loudest possible scanner sounds to safe levels for the duration of the scan. To achieve this noise reduction, we combined three forms of hearing protection: first, silicone earplugs (Mack’s Pillow Soft Kids Silicone Earplugs) were inserted into the opening of each ear and expanded over the ear canal (they are tacky and so stay in place better than foam plugs). Although extremely effective and the best option we have found, these do take a couple of minutes to apply and can cause the infant to become fussy; if this occurred during the orientation session, we typically provided parents with a sample to practice with at home. Second, soft foam cups with hydrogel adhesive at the rim (MiniMuffs, Natus) were placed over the earplugs and attached to the outer ear. Third, MRI-safe passive circumaural headphones (MRI Pediatric Earmuffs, Magmedix) were placed over the foam cups. These three layers were intended to provide redundancy, so that if the earplugs became dislodged the headphones would provide adequate protection and vice versa. Whenever there was a break in scanning, we verified that the hearing protection was intact and re-applied if not. As anecdotal evidence that the final sound level was comfortable, infants did not startle when scans started and sometimes fell asleep. We initially piloted other types of hearing protection outside of the scanner, such as stickers over the tragus, or circumaural headphones that were held around the head by an elastic band, but found that they could not be applied securely and were more prone to failure. The parent(s) in the scanning room were given traditional hearing protection throughout the scan: foam ear plugs and circumaural headphones. Although the scanner is loud, we were able to talk over the noise during the scan to communicate with parents.

### Scan sessions

We scheduled a scan as soon as possible after the orientation. We left it up to the parent to decide when in the day they thought their child would be best able to participate. They tended to choose times after napping and feeding (often in the morning), though work and childcare constraints and scanner availability also influenced scan time.

Upon arrival, we performed extensive metal screening. Every session, parents filled out a metal screening form for themselves and on behalf of their child, which checked for a medical or occupational history of metal in or on their body. After removing clothing, shoes, and jewelry with metal and emptying their pockets, the parent carried the child into a walk-through metal detector. If the detector sounded, often because of a small amount of metal on the parent (e.g., a clasp), one of the experimenters walked with the infant through the metal detector. To double check the child, we passed a high-sensitivity metal-detecting wand (Adams ER300), able to find small internal or ingested metal, over their front and back. We additionally asked the parent if they had seen their child eat anything metallic in the past few days and did not proceed if that was a possibility. Finally, we encouraged parents to bring metal-free toys, pacifiers, and blankets into the scanner to comfort the child; we screened all of these items with the wand before taking them in.

The parent(s) and infant entered the scanner room with one or two experimenters. The hearing protection was put on the infant by one of the experimenters while the other experimenter and parent(s) entertained the child. The infant was then placed on the scanner table. The infant’s head was rested on a pillowcase covering a foam pad in the bottom half of a 20-channel Siemens head/neck coil. The headphones formed a somewhat snug fit reducing the amount of lateral head motion possible, but no additional padding or restraint was used around the head. The infant’s body from the neck down was rested on a vacuum pillow filled with soft foam beads (S & S Technology) covered by a sheet. The edges of the vacuum pillow were lifted and loosely wrapped around the infant to form a taco shape, and the air was pumped out of the pillow until it conformed to the infant’s body shape. This prevented the infant from rolling off the table or turning over, while also reducing body motion during scans. On occasion, and with the recommendation of the parent(s), we swaddled young infants in a muslin blanket before placing them on the vacuum pillow. Overall, however, we found that infants tended to move their head and body less when snug but not constricted. The infant’s eyes were covered by an experimenter’s hand while the head was isocentered in the scanner with a laser.

Unlike typical fMRI studies, we did not attach the top half of the head coil. This decision was made for several reasons. It would have obscured the view of the infant’s face from outside the bore, limiting the ability of the parents and experimenters to monitor the infant. The top of the coil would have also blocked the line of sight between the infant and the ceiling of the bore, interfering with our eye-tracking camera and preventing infants from seeing the entire screen projected on the bore ceiling. We also worried that covering the infant’s face would induce unnecessary anxiety and that the hard plastic of the top coil presented an injury risk if the child attempted to raise their head (which occurred regularly).

**Fig. 7:**
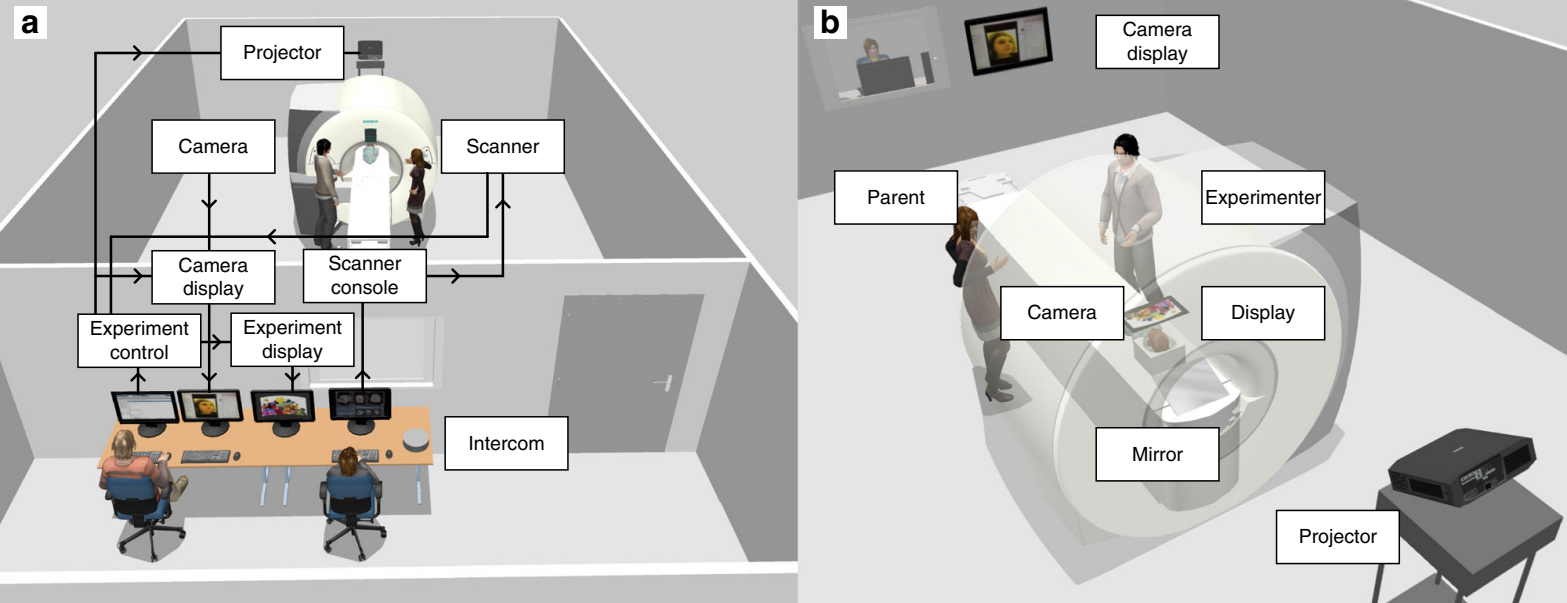
Schematic of the scanning environment. **a** Overview of the setup and wiring diagram of the equipment and communications. **b** Key elements inside the scanner room as well as a view of the screen projected onto the ceiling of the bore. The camera is adjacent to the screen projection. The video feed is depicted on a screen on the wall but could be shown a monitor through the window. 3D rendering created using Sweet Home 3D from Sweet Home 3D assets shared under a Free Art 1.3 licence and CC-BY 3.0 license. Additional assets are from Trimble 3d Warehouse in accordance with their license. All rights reserved.

Fig. 7 illustrates the configuration of the research team during infant scanning. We found that it was critical for an experimenter with exceptional bedside manner to remain inside the scanner room adjacent to the parent(s). They monitored and supported infant comfort using a combination of physical contact, viewing the infant in the bore directly, and watching them on the video camera. They additionally provided explanations and directions to the parent(s). This experimenter also adjusted and focused the video camera (12M-i camera, MRC Systems) that was attached to the ceiling of the bore in order to get a clear view of the infant’s eyes. The video feed from the camera was streamed to a monitor, which further helped the experimenter and parent(s) in the scanner room monitor the infant. We placed the monitor against the glass of the window between the control room and scanner room, though as an alternative the video feed can be displayed on a screen or projector inside the scanner room.

The experimenter in the scanner room communicated with the research team in the control room about which tasks to run, when to start and stop scans, and how data quality was looking. For Cohort I, the control room spoke over an intercom to the experimenter in the scanner room wearing headphones (Slimline, Siemens), who, in turn, communicated back with the control room using hand signals visible through the window. For Cohort II, a two-way communication system was used, allowing the experimenter in the scanner room to listen to the control room over headphones (OptoActive II, Optoacoustics) and speak to them through a microphone affixed with velcro tape to the front of the scanner bore (FOMRI III, Optoacoustics). The two experimenters in the control room operated computers and equipment. One experimenter controlled the Siemens console computer (e.g., setting up sequences, adjusting alignment, monitoring data quality) and was responsible for communicating with the experimenter in the scanner room. The other experimenter controlled another computer running experimental tasks, the eye-tracker, and the vacuum pump.

### Experiment menu

Given the unpredictability of working with infants, we developed an experiment menu software system that provides complete flexibility in running cognitive tasks during fMRI. This system dynamically generates and executes experimental code in Psychtoolbox^37^ for MATLAB (MathWorks). The experimenter could easily navigate to an experiment from a library of tasks and choose a specific starting block (allowing tasks to be interrupted and resumed), or they could review the progress of an experiment so far. The code coordinated all timing information, receiving and organizing triggers from the scanner, and starting and stopping eye-tracker recordings. After each block ended, there was a short delay before the next block started, allowing the experimenter to determine whether to continue to the next block in the same task, switch to a new task, or stop altogether. It was also possible to rapidly switch to a movie at any point, which we showed during anatomical scans, to regain infant interest and attention, and for certain naturalistic experiments.

This integrated and semi-automated framework for experiments and eye-tracking reduces the burden on experimenters and the possibility of manual errors during already complex procedures. The ability to switch between tasks efficiently within a single ecosystem reduced downtime where the infant was lying in the scanner without any task. This not only increased the amount of time during which usable fMRI data could be collected, but also reduced fussing out that was more likely to occur when nothing was on the screen. Although we developed this system for infants, it could also be used for patient testing and other special populations who present similar complications.

The experiment menu system can flexibly incorporate a range of cognitive tasks. Any task that can be designed in Psychtoolbox can be ported to this system, regardless of consideration to response inputs, experiment duration, display parameters, or other factors. To help users understand how the experiment menu works, we have provided two sample experiments in the software release that interact with the system in different ways and can be easily modified.

### Eye-tracking

Different types of eye-trackers can be integrated with our software architecture (e.g., EyeLink from SR Research, iViewX from SMI). For Cohort I, we used the frame-grabber capabilities of iViewX eye-tracker software to receive and record input from the MRC video camera. This set-up required manually starting and stopping eye-tracking. For Cohort II, the same camera fed a dedicated eye-tracking computer via a frame-grabber (DVI2USB 3.1, Epiphan). This additional computer ran Python code to save every video frame with a time stamp and was connected to the main experiment computer via ethernet to receive messages, start and stop recording, and perform handshakes. These frames were corrected for acquisition lag and manually coded offline by two or more raters.

To facilitate manual gaze coding, the provided software includes a tool to display relevant video frames offline and convert coded responses into a format compatible with the analysis pipeline. The system was designed to make this laborious task more efficient, allowing coders to quit and resume, accelerate their coding speed, and adjust their FOV. Response code options are flexible (e.g., eyes open vs. closed, fixation vs. saccade, gaze left/center/right, etc.) and could even be used for non-eye behavior (e.g., head motion). This tool also computes coding reliability across raters.

Manual gaze coding is a time-intensive and somewhat crude procedure compared to modern eye-tracking standards. However, in our experience, the currently available automated eye-tracking systems are infeasible for infant fMRI. Most such systems used in infant behavioral or adult fMRI studies require a calibration phase in which visual transients appear in different locations. In our experience, infant gaze is captured with insufficient reliability in the scanner to make these calibrations viable. Additional problems, such as needing to adjust the infrared emitter and FOV whenever the infant moves, make automated eye-tracking difficult to manage during a protocol that is already challenging. However, we remain optimistic that computer vision algorithms may be capable of automating some of the gaze coding humans currently perform in our protocol^38^, reducing the burden and potentially increasing the reliability of this approach.

### Ceiling projection

We developed a stimulus display system for infants. When using a typical rear-projection system for fMRI, the stimulus is projected on a screen at the back of the bore and the screen is viewed on an angled mirror attached to the top of the head coil. As a result, the stimulus usually covers a small part of the visual field and requires a specific vantage point through the mirror. We could not be sure that these displays would grab the attention of infants. At a more basic level, mirrors may confuse or distract infants. Another approach could be to use a goggle system^22^, which guarantees that the infant can see the stimulus. However, it is hard to monitor the infant with such a system and taking it off can be disorienting.

Instead, we projected visual stimuli onto the ceiling of the scanner bore over the infant’s face. We mounted a projector (Hyperion, Psychology Software Tools) approximately six feet high on the back wall behind the scanner, tilted downward to project at the back of the bore. A large mirror placed in the back of the bore behind the scanner table at a low angle reflected the image up onto the bore ceiling, as shown in Fig. 7b. This provided a high resolution display (1080p) and wide FOV stimulation (approximately 115 degrees of visual angle). The thrown image suffered from keystone, elliptical, and stretching geometric distortions, as a result of the angled projection, reflection, and curved bore, but these were corrected automatically in software by a preset screen calibration in the experiment menu code. For Cohort I, we projected directly on the white plastic surface of the bore ceiling. For Cohort II, we taped a piece of white paper to the ceiling to hide the plastic grain. We believe that this large and direct display kept the infants engaged and was natural for them to view. It also gave the parent(s) and experimenters a clear view of the child’s face and allowed for seamless video eye-tracking without calibration. The experiment menu code can be used to set-up and calibrate ceiling projection, but is also compatible with other display types, including rear-projection screens or goggles; tools are included to equate stimulus sizes across display formats.

### Tasks

We report visual responses from fMRI data combined across a variety of stimuli used in several ongoing experiments, including blocks of: looming colorful fractals (Cohort I: 11 runs, Cohort II: 14 runs; 14.6° max size), looming toy photographs (Cohort I: 7 runs, Cohort II: 6 runs; 8°), looming face photographs (Cohort I: 5 runs, Cohort II: 0 runs; 8°), and moving shapes (Cohort I: 9 runs, Cohort II: 6 runs; 10–15°).

Each experimental task was designed to be short, entertaining, and modular. Task blocks generally lasted less than 40 s, though sometimes were longer, as in the case of movies. The tasks used visual effects to maintain attention, including fast motion and onsets (e.g., looming), high-contrast textures, bright colors, and relevant stimuli (e.g., faces). Our goal for each session was to obtain up to three full experiments worth of data, which we achieved on occasion. However, the tasks were designed and counterbalanced internally to provide useful data even when incomplete. Infants sometimes found a given task boring and began fussing or moving, and in such circumstances, we adapted by changing to a new experiment (sometimes later returning to the original experiment). We found that fussing out of one task did not predict that the child would fuss out of other tasks, hence being able to switch tasks within participant increased data yield. The menu system automatically handled timing, scheduled rest periods between blocks/tasks, counterbalanced conditions, and tracked stimulus order and novelty.

At some point during the session, typically after at least one attempted experiment, we collected an anatomical scan. This scan was used for registration of functional data and alignment to anatomical templates. Obtaining a high-quality scan was especially difficult because the infant had to remain still for the entire duration of 3.25 min (whereas for functional scans, discrete motion only impacted a small number of 2-s volumes). If the infant was awake, we did our best to keep them entertained with either a compelling visual task (e.g., fireworks appearing in different parts of the display) or a movie (e.g., Daniel Tiger, Sesame Street). If asleep, we blanked the screen. We attempted as many anatomical scans as needed to obtain one of sufficient quality (and as time allowed), though often succeeded in one try.

### Fussiness

Our goal was to make the session as fun and as enjoyable as possible but it was inevitable that some infants got fussy. In our experience, this happened most often at one of three stages: when putting the hearing protection on the infant, when first laying the infant down on the scanner table, and/or when the infant got bored with a task. It was rare that other events, such as the scanner starting, triggered unhappiness. In fact, many infants seemed to be soothed by the scanner sounds and vibrations, and some enjoyed the visual displays so much that they fussed only when removed from the scanner. We did find that talking (loudly) to the infant between scans and patting or holding their hands was soothing. Neither the parents nor experimenters climbed into the bore with the infant. We did not encourage this to avoid distracting the infant and inducing motion or potential confounds. Without such distraction, we found that infants on their own (within arm’s reach) quickly became enraptured by the visual display. We did allow infants to use pacifiers, soothers, bottles, or blankets while in the scanner, which generally had a soothing effect. Although the movement of their jaw while sucking on a pacifier could add noise, this noise was less than that from the motion of an unhappy infant and outweighed the negative impact of otherwise collecting much less data in some sessions.

If a fussy infant could not be soothed or attempted to roll over or climb out, or if the parent(s) asked for a break, we took the infant out of the scanner until they were calm again and ready to resume. The parent would often nurse the infant or give them a bottle or snack, and would change their diaper if needed. In some cases, we had to start and stop 3–4 times before the infant became sufficiently comfortable to provide high-quality data. When the infant had completed all planned experiments, had been in the scanner room for an hour, became too fussy, or fell asleep for a long time (after we completed anatomical scans) we ended the session. In addition to monetary compensation for the family’s time and travel, and a board book for the infant, we also printed a 3-D model of the infant’s brain whenever possible (using Ultimaker 2+ to print surface reconstructions from FreeSurfer^39^). We encouraged families to come in for multiple sessions, and many were happy to do so, generally with one month or more between visits.

### Inter-session variability

Our protocol was designed to be flexible across participants and within-participant across sessions, in order to account for infant temperament, reaction to tasks, and non-experimental disruptions (e.g., play, feeding, diapers, sleeping, etc.). This flexibility has the benefit of increasing retention and maximizing the amount of data and number of tasks that can be administered per session. However, it comes at the cost of increased variability across sessions, which complicates the analysis and interpretation of data. For example, the sequence of tasks is hard to control, raising the possibility of order effects and habituation.

We have taken several steps to attempt to mitigate this variability: First, we do our best to avoid introducing unnecessary variability. We try to be as consistent as possible across sessions about the scanner environment (location, waiting room, toys, etc.), personnel (parents and researchers), head coil and padding, hearing protection supplies and application, MRI sequences, presentation stimuli, and preprocessing parameters. Second, all tasks are designed using within-subject manipulations for which there are no parsimonious accounts about how prior tasks or habituation could drive condition-wise differences. Third, we use different categories of stimuli (faces, objects, shapes, cartoons, etc.) and presentation styles (looming, oscillating, dynamic, etc.) across tasks to minimize habituation. Fourth, we present tasks in a pseudo-random order across sessions and participants, which would serve to counterbalance task history effects under ideal circumstances. Finally, it is worth noting that although attempting multiple tasks within a session may pose these complications, collecting multiple measures from a sufficient number of infants makes it possible to test for order effects, habituation, and more theoretically, how different cognitive capacities relate and interact.

### Data acquisition

Infant data were acquired on a 3T Siemens Skyra MRI in Cohort I and on a 3T Siemens Prisma MRI in Cohort II using anatomical and functional sequences (see Supplementary Table 1 for a summary of parameters). For anatomical scans, we used a T1-weighted PETRA sequence in all participants (${\rm{TR}}_{1}$ = 3.32 ms, ${{\rm{TR}}}_{2}$ = 2250 ms, TE = 0.07 ms, flip angle = 6°, matrix = 320 × 320, slices = 320, resolution = 0.94 mm isotropic, radial lines = 30,000). In two young infants, we additionally piloted a T2-weighted SPACE sequence (TR = 3200 ms, TE = 563 ms, flip angle = 120°, matrix = 192 × 192, slices = 176, resolution = 1 mm isotropic). For functional scans, we used a T2*-weighted gradient-echo EPI sequence in all participants (TR = 2000 ms, TE = 28 ms, flip angle = 71°, matrix = 64 × 64, slices = 36, resolution = 3 mm isotropic, interleaved slice acquisition). The FOV covered the whole brain during slice positioning, but occasionally parts of the brain were cropped as a result of head motion, typically the cerebellum and brain stem. We did not use multi-band slice acceleration (common in modern adult fMRI) because of concerns about peripheral nerve stimulation that could not be reported by our preverbal infants. The adult data for SFNR comparison (Fig. 2) were acquired on the same scanner as Cohort I and with the same functional sequence, except that the top of the head coil was attached. We had whole-brain coverage in adults, although the cerebellum and brain stem were cropped in some participants.

### Preprocessing

We developed an efficient analysis pipeline for preprocessing infant fMRI data. This software has been released publicly with this paper and pairs particularly well with the experiment menu system described above. The code is modular and easily editable, while also largely unsupervised. Indeed, any task-based fMRI experiment could in principle be analyzed with this pipeline. Despite the variability noted above in the order and duration of tasks and the amount of data collected per session, many of the consistent aspects of our protocol (e.g., experiment menu system, within-task experimental design and timing, MRI acquisition parameters, etc.) standardize the processing such that it takes less than 2 h on average to run raw infant fMRI data through the pipeline. Supplementary Fig. 1 depicts the overall preprocessing pipeline schematically. To help users learn how this pipeline works, we include extensive documentation in a step-by-step tutorial with the pipeline.

Videos of the infant’s face collected during scanning were blindly coded offline for eye-gaze by 2–7 naive coders, based on task-specific criteria^40^. Tasks that only required fixation (e.g., movie watching) were coded for whether the eyes were on-screen or off-screen/closed. Tasks that involved viewing images on the left and right of the display were coded for the direction of looking. Gaze location was labeled by calculating the modal response across coders for a time window of five video frames (100 ms). A tie in the coding was resolved by assigning the label from the most recent frame that was not a tie. We calculated inter-rater reliability by comparing the consistency of responses across coders.

After the data from the scanner were converted into NIFTI format, we calculated motion parameters for the functional data. The movement behavior of infants was different from adults because it was often punctate, large in magnitude, and unpredictable, rather than slow and drifting. Hence, the best reference volume to use for motion correction might be different in infants and adults. Specifically, rather than using the first, last, or middle time-point, as is typical in adults, we selected the volume with the minimum average absolute euclidean distance from all other volumes (the centroid volume) as the reference. We used FSL^41^ (5.0.9 predominantly) for calculating frame-to-frame translations and identified time-points that ought to be excluded because of motion greater than our threshold (3 mm).

The stimulus and timing information from each task were converted into FSL timing files using a script. Epochs of data (trials, blocks, or runs) were marked for exclusion at this stage if there was excessive motion and/or if the infant’s eyes were off-screen/closed for more than half of the time-points in the epoch or during a critical part of the epoch for the task. Manual exclusions of data were also specified here, such as when the infant moved out of the FOV of the scan. The anatomical data were preprocessed using AFNI’s homogenization tools, combined with other anatomical data if available, and skull stripped (AFNI’s 3dSkullStrip^42^).

If more than one task was tested within a run, we created pseudo-runs in which time-points corresponding to the different tasks were extracted and used to create new run data. This happened more often in Cohort II, in part because we realized between cohorts that we obtained more usable data with less downtime when we scanned continuously rather than stopping arbitrarily when infants finished an experiment. Indeed, Supplementary Table 2 shows that we tended to terminate more runs when an experiment finished in Cohort I than in Cohort II. Centroid volumes and motion exclusions were recomputed for these pseudo-runs, which were then input to the preprocessing analyses as if they were collected as separate runs.

First-level analyses were performed to preprocess each run. We started from FSL’s FEAT but added modifications to better accommodate infant fMRI data. We discarded three burn-in volumes from the beginning of each run. We interpolated any time-points that were excluded due to motion, so that they did not bias the linear detrending (in later analyses these time-points were again excluded). We performed motion correction using MCFLIRT in FSL, referenced to the centroid volume as described above. The slices in each volume were acquired in an interleaved order, and so we realigned them with slice-time correction. To create the mask of brain and non-brain voxels we calculated SFNR^20^ for each voxel. This produced a bimodal distribution of SFNR values reflecting the signal properties of brain and non-brain voxels. We thresholded the brain voxels based on the trough between these two peaks. The data were spatially smoothed with a Gaussian kernel (5 mm FWHM) and linearly detrended in time. AFNI’s despiking algorithm was used to attenuate aberrant time-points within voxels.

We registered each participant’s functional volumes to their anatomical scan using FLIRT in FSL with a normalized mutual information cost function. However, we found that this automatic registration was insufficient for infants. With this as a starting point, we used mrAlign (mrTools, Gardner lab) to perform manual registration (6 degrees of freedom). One functional run from each session was aligned to the anatomical scan and then each additional run was aligned to the anatomically aligned functional data, all in native resolution. This process was repeated as necessary to improve alignment.

As with registration of functional data to anatomical space, a combination of automatic and manual alignment steps (9 degrees of freedom) were usually needed to register the anatomical scan to standard space (using Freeview from FreeSurfer^39^). The standard space for each infant was chosen to be the infant MNI template closest to their age^43^. These infant templates were then aligned to the adult MNI standard (MNI152 1 mm). This alignment step into adult standard space was performed for two reasons. First, it ensured that data were analyzed in a common space across the age span (even if the detailed anatomy does not fully correspond). Second, it allowed us to define anatomical ROIs, which are based on templates in adult space. For the present work, alignment to standard space was performed only after generating the statistic maps in native resolution.

After preprocessing and registration, the data can be reorganized into individual experiments. We did not perform this step for the data reported here because we wanted to include as much data as possible. That is, we analyzed runs from multiple visual tasks and collapsed across these runs for session-wise analyses. Nevertheless, we describe this step in detail below because it has been implemented in our shared software pipeline and will generally be useful for future studies of a particular task. Reorganizing data into individual experiments helps account for the fact any given experiment could be spread across multiple runs (e.g., because of breaks or fussiness). Pseudo-runs of the same task (extracted from runs with multiple tasks) and entire runs in which only that task was tested would be concatenated into a single experiment dataset per participant. This dataset can then be checked for counterbalancing across task conditions to prevent biases or confounds related to run number or time. For instance, if an experiment has two conditions, an equal number of epochs from each condition can be selected per run. The voxel time-series for the usable epochs should be *z*-scored within run prior to concatenation, to eliminate generic run-wise differences in the mean and variance of BOLD activity. The corresponding timing and motion information would also get concatenated. These datasets can then be used as inputs for analyses of individual experiments.

### Analysis

SFNR was calculated from the raw infant and adult fMRI data. For each voxel in the brain mask, the mean activity was divided by the standard deviation of the detrended activity. The detrending was performed with a second-order polynomial to account for low-frequency drift^20^. We analyzed data from 16 adults with one run each (16 total) containing 260 volumes and from 19 infant sessions with 1–6 runs each (64 total) containing 6–335 volumes (*M* = 112.3). One run (6 TRs) was excluded because of severe aliasing. To quantify posterior-to-anterior changes in signal, SFNR was estimated for each coronal slice. These slices were taken along the y-axis of the acquisition slab, and thus were not precisely aligned with the posterior-to-anterior axis in the reference frame of the head or brain. The average SFNR for each coronal slice (with at least 1000 brain voxels) was computed by sampling the SFNR values of 1000 voxels in that slice. We used this subsampling approach to control for the number of voxels used in averaging across infant and adult brain sizes. The coronal slices were then median split into posterior and anterior halves, which served as a within-subject factor in a repeated measures ANOVA of SFNR, along with age group (infants or adults) as a between-subject factor. Note that since infant brains are smaller on average this means that fewer slices of their brains will be included, particularly at the posterior and anterior edges. Sampling fewer voxels (e.g., 100) and thus including more slices led to consistent results.

To understand how head motion impacted SFNR, the translational motion between each TR was first computed using MCFLIRT in FSL and averaged across the run. Participants were selected as low-motion if they had average translational motion of less than 0.2 mm across a run. The relationship of motion to SFNR was quantified by correlating the average motion for each run with the average SFNR across all coronal slices for each run. Both average motion and average SFNR were non-normal, with long tails, and so we estimated the non-parametric Spearman’s rank correlation coefficient (*ρ*). The *p*-value was computed by resampling participants with replacement 10,000 times, recomputing *ρ* for each sample, and then calculating the proportion of samples with a positive sign (as the true relationship was negative).

The analysis of visual evoked activity included all of the runs/pseudo-runs that contained at least two task blocks, excluding epochs that were not usable because of motion, eye-gaze, or inappropriate data type (e.g., movie watching or resting state). We fit a univariate GLM across the whole brain, modeling the response with a task regressor convolved with a double-gamma hemodynamic response function as the basis function. Nuisance regressors were specified for motion relative to the centroid volume (six degrees of motion, including x, y, z translation and yaw, pitch, roll rotation), as well as a single time-point regressor for each excluded time-point. The *z*-statistic map for the task regressor in every run was then aligned into adult standard space.

For the ROI analyses, we defined anatomical ROIs for V1, LOC, and A1 using the Harvard-Oxford atlas. For each ROI and run/pseudo-run, we quantified the proportion of voxels with a *z*-score corresponding to *p* < 0.05. The significance of these proportions across runs relative to chance (0.05) was calculated with bootstrap resampling^44^. We sampled with replacement the same number of run proportions from each ROI 10,000 times to produce a sampling distribution of the mean (or mean difference between ROIs). The *p*-value corresponded to the proportion of resampling iterations with mean below chance (or below zero for differences between ROIs). For exploratory voxelwise analyses, we used randomise in FSL to compute a *t*-statistic in each voxel and then applied an uncorrected threshold of *p* < 0.005.

In the analyses above, runs/pseudo-runs were treated as independent samples, to assess the statistical reliability of visual responses at the run level. However, there was often more than one run per session, allowing us to additionally examine reliability at the session level. Hence we repeated the same analyses above after concatenating all runs/pseudo-runs within the session so that there was only one GLM and set of voxelwise *z*-scores per participant. Despite the smaller number of values entered into the final statistical analyses, the results were largely unchanged (Supplementary Fig. 5), likely the result of including more data in computing each value and thus obtaining cleaner estimates.

To explore how preprocessing decisions affected these results, we repeated the univariate analyses above while varying several parameters in our pipeline one at a time: the threshold for excluding individual time-points based on the head motion; whether to exclude time-points after instances of motion (after a brain moves there is an imbalance in net magnetization of each slice that can take several seconds to correct); the FWHM of the smoothing kernel; whether to exclude components of the data extracted with independent components analysis (ICA, MELODIC in FSL) that correlate with the six motion parameters (we varied the minimum correlation threshold for regressing out components); whether to use voxelwise despiking to remove aberrant data that can result from motion in voxels near the skull or ventricles; and whether to include the temporal derivatives of the regressors in the GLMs, to account for latency differences.

Some of the preprocessing decisions (e.g., motion threshold) affected our time-point exclusion procedure and, in turn, the amount of data retained. To calculate these retention rates, we excluded individual time-points because of the above-threshold motion and entire blocks when the majority (>50%) of its time-points were excluded. That is, time-points that are themselves usable but part of an unusable block become unusable. For reference, 100% would mean that in runs with at least two usable task blocks, all time-points from all participants were usable. Even when no motion exclusions are performed, some blocks will still not be usable due to eye exclusions or because we terminated the block prior to completion. Note that these rates do not account for usable data that were excluded from the analysis of visual responses because the corresponding task was unsuitable for estimating evoked responses (e.g., movie watching).

To compare across parameters for each type of preprocessing, we ran a linear mixed model with condition (parameter) as a fixed effect and run (or session) as a random effect. This approach was chosen instead of a repeated measures ANOVA in order to deal fairly with missing data in cases where a run/session was excluded (e.g., because there were no longer two usable blocks). The Wald Chi-Square test was used as an omnibus test to determine whether there were any significant differences in the model, and simple effect comparisons were used to test our default parameter setting against the other options.

### Reproducibility

Cohort II served as a replication and generalization sample of Cohort I. Although the ages and precise composition of tasks differed between cohorts, the pattern of results was identical, with significant visual evoked activity in V1 and LOC, but not in A1.

### Reporting summary

Further information on research design is available in the Nature Research Reporting Summary linked to this article.

## Supplementary information

Supplementary Information

Reporting Summary

Description of Additional Supplementary Files

Supplementary Movie 1

## Data Availability

The anatomical and functional MRI data from both cohorts are publicly available [10.5061/dryad.8gtht76k3]. The two included datasets are sufficient to recreate Fig. 2 (infants), 3, 4, 5, 6; Supplementary Figs. 3 (infants), 4 (infants), 5, 6, 7; and Supplementary Tables 2, 3, 4. Code is provided to generate these images from the shared data. A reporting summary for this Article is available as a Supplementary Information file. Source data are provided with this paper.

## Source data

Source Data

## Code availability

We have released general software packages that can be used flexibly by other researchers to run new infant fMRI studies, including for data acquisition [https://github.com/ntblab/experiment_menu] and data analysis [https://github.com/ntblab/infant_neuropipe]. We have also shared the specific instantiation of the code needed to generate the results reported in this paper [https://github.com/ntblab/infant_neuropipe/tree/methods_validation].
