# Supplementary material for: Re-imagining fMRI for awake behaving infants
Source: Nat Commun. 2020 Sep 9;11:4523. doi: 10.1038/s41467-020-18286-y (PMC7481790; doi:10.1038/s41467-020-18286-y)
Supplement: Supplementary file 1 — Supplementary Information [file 41467_2020_18286_MOESM1_ESM.pdf]

Supplementary Information

Re-imagining fMRI for awake behaving infants

Ellis, C. T., Skalaban, L. J., Yates, T. S., Bejjanki, V. R., Córdova, N. I., & Turk-Browne, N. B.

Corresponding author: Nicholas B. Turk-Browne, nicholas.turk-browne@yale.edu

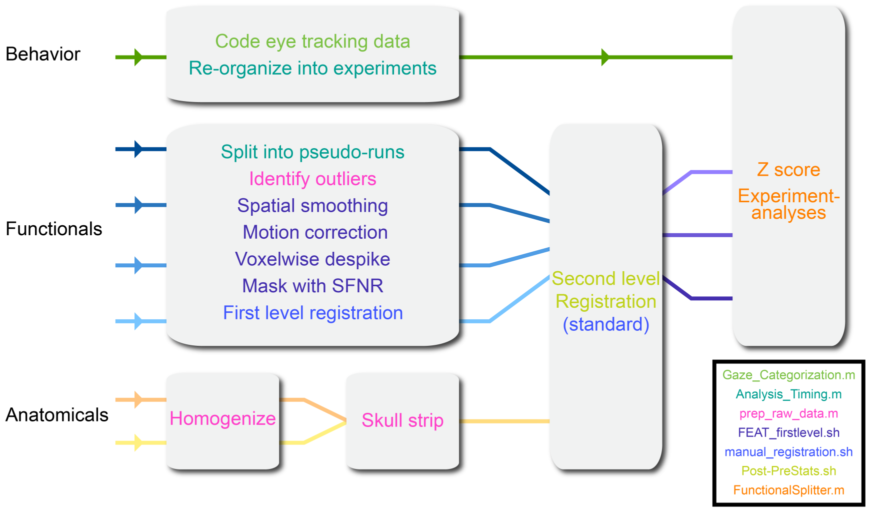

Supplementary Figure 1: Schematic of the analysis pipeline. This pipeline allows for relatively standardized and automatic processing of infant fMRI data, despite variability across sessions in the amount of data, quality of data, and number and types of tasks. Color codes correspond to the scripts utilized to perform each operation.

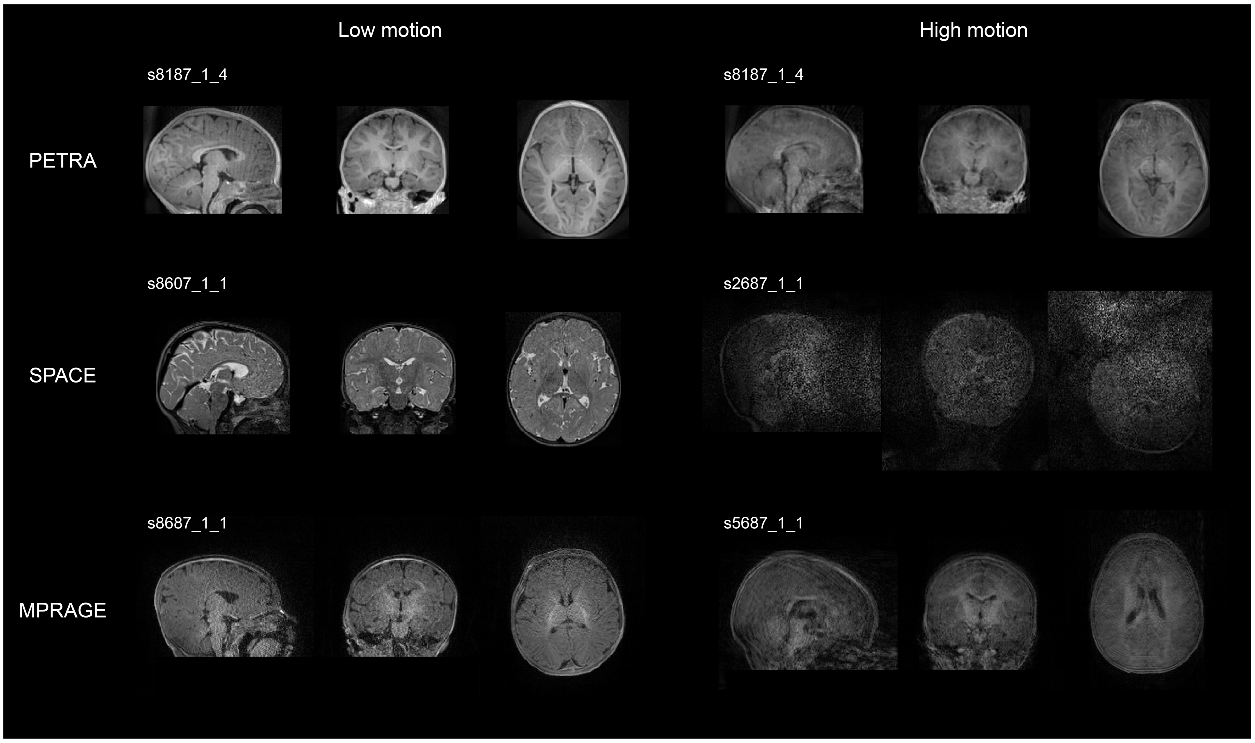

Supplementary Figure 2: Examples of anatomical scans from different MRI sequences. Left side shows low motion anatomical scans, right side shows high motion scans. Refer to Supplementary Table 1 for the parameters of each scan.

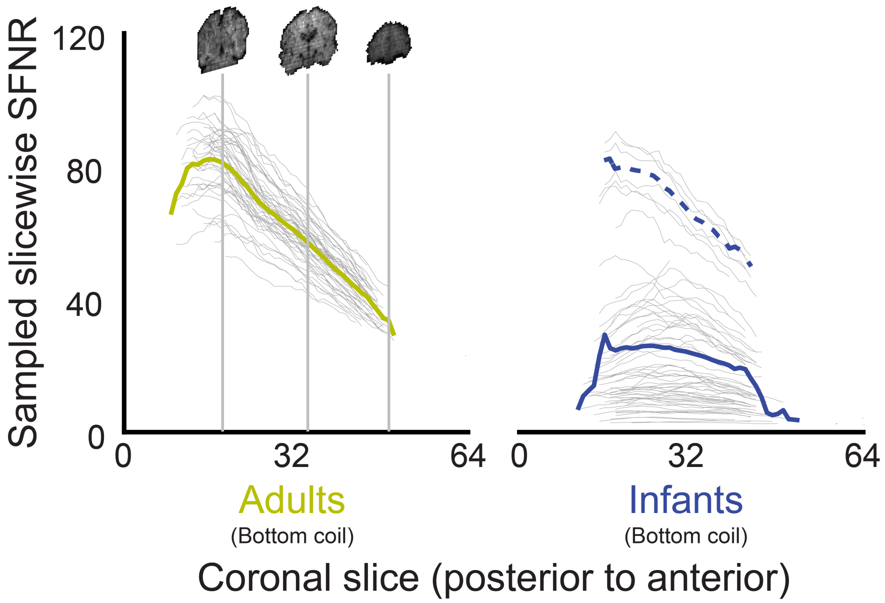

Supplementary Figure 3: Infant versus adult signal-to-fluctuation-noise ratio (SFNR) when the top of the head coil is not connected. The infant data are identical to Figure 2 (solid blue = mean, dashed blue = low-motion infants). The adult data (48 sessions, 48 runs, containing 93-152 TRs, M=113.1) were collected for an unpublished study on a Siemens Prisma with equivalent sequence parameters to the infant data, except TE = 32ms (vs. 28ms) and 34 slices (vs. 36). SFNR was higher in adults (M=62.0) than infants (M=23.6) over the whole brain ($F(1,109)=174.18$, $p<0.001$). SFNR was higher in the posterior (M=46.1) than anterior (M=34.3) half of the brain ($F(1,109)=521.83$, $p<0.001$). The drop was smaller in infant than adult participants, in both proportional (adult M=0.69, infant M=0.92, Welch's $t(80.6)=13.53$, $p<0.001$) and absolute terms ($F(1,109)=366.77$, $p<0.001$). Source data are provided as a Source Data file.

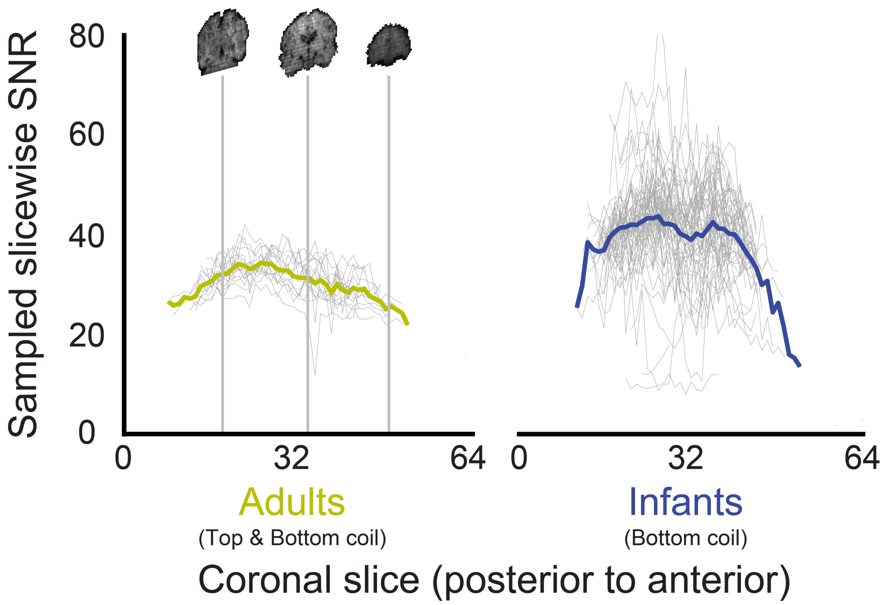

Supplementary Figure 4: Slicewise signal-to-noise ratio (SNR) across the brain for adult and infant data. Adult data were collected with both the top and bottom of the head coil attached, whereas infant data had only the bottom head coil attached. Coronal slices of the centroid volume that contained at least 1000 brain voxels and 500 non-brain voxels were used. To calculate SNR in each slice, the average of a random sample of 1000 brain voxels was divided by the standard deviation of a random sample of 500 non-brain voxels (Triantafyllou, et al., *NeuroImage*, 2005; Weisskoff, *Magnetic Resonance in Medicine*, 1996). SNR was numerically higher in adults (M=31.1) than infants (M=22.7) over the whole brain ($F(1,77)=3.26$, $p=0.075$). SNR was higher in the posterior (M=26.5) than anterior (M=22.3) half of the brain ($F(1,77)=25.91$, $p<0.001$). The drop was not significantly different between infants and adults, in neither proportional (adult M=0.91, infant M=0.88, Welch's $t(76.8)=-1.34$, $p=0.183$) nor absolute terms ($F(1,77)=0.67$, $p=0.416$). Source data are provided as a Source Data file.

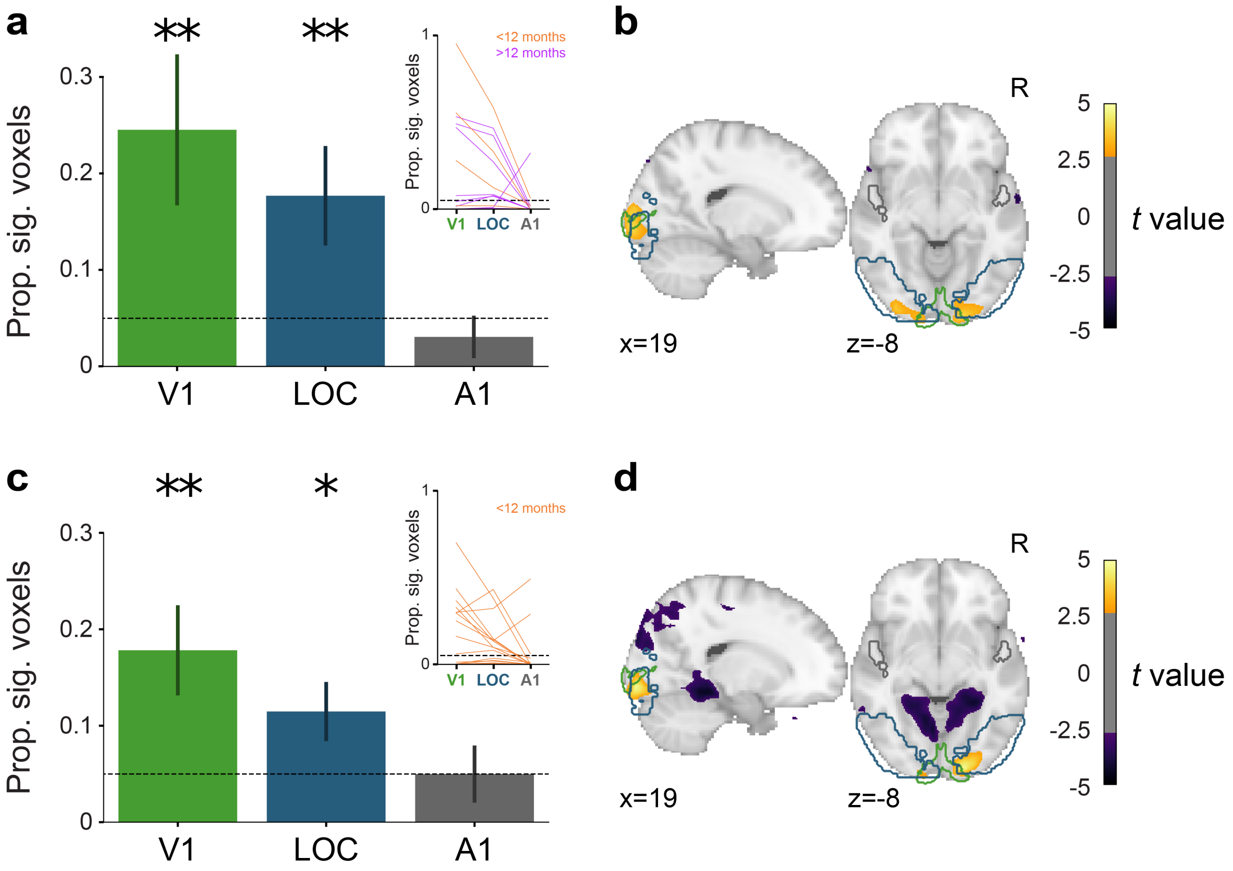

Supplementary Figure 5: Visual evoked activity for each session from Cohorts I and II. Rather than analyzing each run, here we analyze each session by concatenating the usable blocks from all runs within a session. a) In Cohort I (N=14), the proportion of voxels showing significant visual responses within session in V1 (*M*=0.25 [*SD*=0.29], *p*=0.005) and LOC (*M*=0.18 [*SD*=0.19], *p*=0.005) was greater than in A1 (*M*=0.03 [*SD*=0.08], *p*=0.424; V1>A1 in 11/14 sessions, *p*=0.008; LOC>A1 in 12/14 sessions, *p*=0.013); V1 was also greater than LOC (7/14 sessions, *p*=0.014). Inset: change in proportion of significant voxels across the ROIs for each run for infants younger (orange) or older (magenta) than a year old. b) *t*-value for voxels across the whole brain showing reliable responses across sessions in Cohort I (*p*<0.005, uncorrected). c) In Cohort II (N=18), the proportion of voxels showing significant visual responses within session in V1 (*M*=0.18 [*SD*=0.20], *p*=0.002) and LOC (*M*=0.11 [*SD*=0.13], *p*=0.015) was greater than in A1 (*M*=0.05 [*SD*=0.13], *p*=0.890; V1>A1 in 9/18 sessions, *p*=0.004; LOC>A1 in 15/18 sessions, *p*=0.038); V1 was also greater than LOC (7/18 sessions, *p*=0.022). Inset: change in proportion of significant voxels across the ROIs for each run (all infants younger than a year old). d) *t*-value for voxels across the whole brain showing reliable responses across sessions in Cohort II (*p*<0.005, uncorrected). Data are presented as mean values +/- between-session SEM. Source data are provided as a Source Data file.

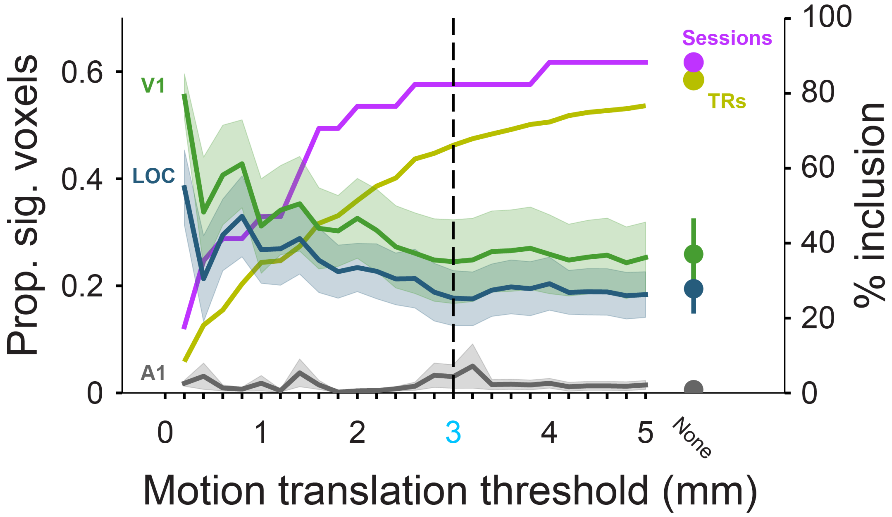

Supplementary Figure 6: Proportion of significant voxels ($p<0.05$) across different motion translation thresholds in V1 (green), LOC (blue), and A1 (grey) for the session-wise data, akin to Figure 4. The dashed line indicates the motion threshold that was used as the default. 'None' indicates the results when no motion threshold was applied. The righthand y-axis reports the proportion of included TRs in mustard and the proportion of runs included in purple (out of 17 sessions with a run containing at least 2 blocks). Note that regardless of the motion threshold, some blocks/runs were excluded because the infant's eyes were closed. Data are presented as mean values +/- between-run SEM as shaded area. Source data are provided as a Source Data file.

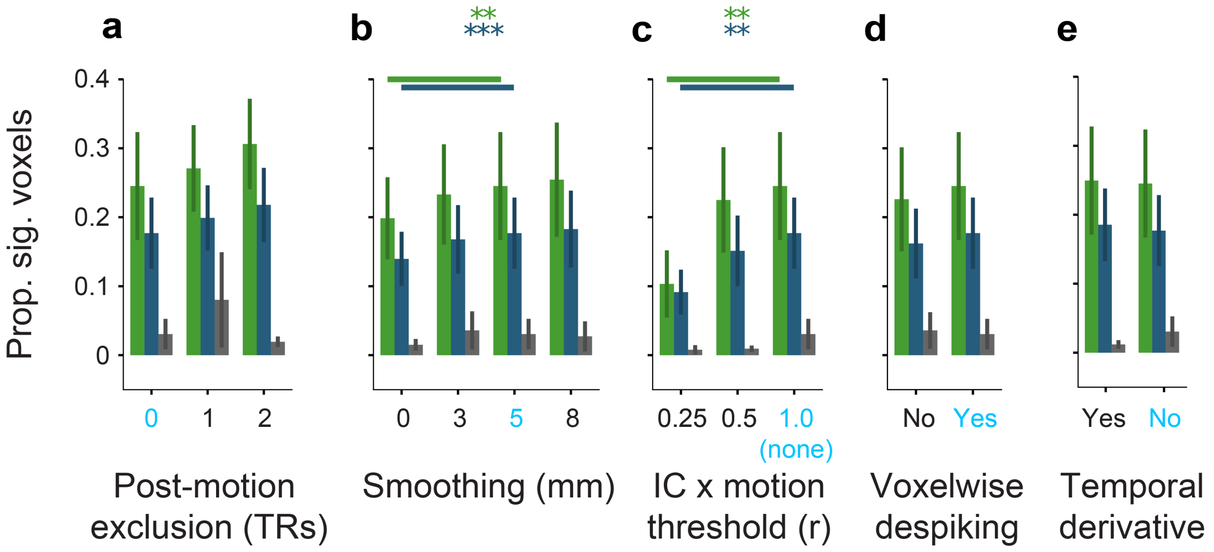

Supplementary Figure 7: Comparison of the proportion of voxels in each ROI showing significant visual responses ($p<0.05$) after various preprocessing decisions for session-wise analyses. Values are separately shown for V1 (green), LOC (blue), and A1 (gray). Parameters are identical to those reported in Figure 5. a) No significant difference in V1 ($\chi^2(2)=0.31$, $p=0.858$), LOC ($\chi^2(2)=0.28$, $p=0.868$), or A1 ($\chi^2(2)=2.02$, $p=0.364$), N=14, 13, and 12 for 0, 1, and 2 time-points removed, respectively. b) Significant difference in V1 ($\chi^2(3)=12.43$, $p=0.006$; 0 vs. 5mm: $t(42.00)=-2.75$, $p=0.009$) and LOC ($\chi^2(3)=16.69$, $p<0.001$; 0 vs. 5mm: $t(42.00)=-3.25$, $p=0.002$), but not A1 ($\chi^2(3)=3.36$, $p=0.340$), N=13. c) Significant difference in V1 ($\chi^2(2)=13.74$, $p=0.001$; 0.25 vs. 1.0: $t(28.00)=-3.43$, $p=0.002$) and LOC ($\chi^2(2)=12.81$, $p=0.002$; 0.25 vs. 1.0: $t(28.00)=-3.49$, $p=0.002$), but not A1 ($\chi^2(2)=1.82$, $p=0.403$), N=13. d) Marginal difference in V1 ($\chi^2(1)=2.73$, $p=0.098$) and LOC ($\chi^2(1)=2.94$, $p=0.087$), but not A1 ($\chi^2(1)=0.62$, $p=0.430$), N=13. e) Marginal difference in V1 ($\chi^2(1)=3.55$, $p=0.059$) and LOC ($\chi^2(1)=3.07$, $p=0.080$), but not A1 ($\chi^2(1)=1.14$, $p=0.285$), N=13. Significance of one-tailed Chi-square test for omnibus linear mixed model: *=$p<0.05$, **=$p<0.01$, ***=$p<0.001$. Significant two-tailed simple effects differences between our chosen parameter setting (in blue) and other settings indicated by a bold line ($p<0.05$). Data are presented as mean values +/- between-session SEM. Source data are provided as a Source Data file.

Supplementary Table 1. Parameters of MRI sequences used in this study and released in the shared datasets. Note that the first several participants had an EPI with a TR of 1.5s and 27 slices. Parameters are included for two additional anatomical sequences for completeness but only two participants produced usable SPACE scans and no participants produced usable MPRAGE scans. Refer to Supplementary Figure 2 for examples of the different anatomical sequences. Refer to Supplementary Movie 1 for examples of functional data with different amounts of motion.

Type	Description	TR (ms)	TE (ms)	Flip angle	Voxel size (x,y,z or isotropic)	FOV (mm)	Slices	BW (Hz/Px)	IPAT	Acquisition time
Scout	T1w localizer	3.15	1.37	8	1.6 x 1.625 x 1.625mm	260	128	540	3	0:14
Functional	T2*w EPI	2000	28	71	3mm, isotropic	192	36	1562	2	33:28 (max)
Anatomical	T1w PETRA	3.32, 2250	0.07	6	0.9375mm, isotropic	300	320	401	NA	3:08
Anatomical	T2w SPACE	3200	563	Variable	1mm, isotropic	192	176	744	2	1:47
Anatomical	T1w MPRAGE	2400	2.42	8	1mm, isotropic	192	176	210	4	3:06

Supplementary Table 2. Full retention data for Cohorts I and II, expanding on what is reported in Figure 1. *Ppt* is the anonymized participant ID. *Age* is participant age (months). *Sex* is participant's assigned sex. *Func min* is usable task time in minutes. *Asleep min* is time infant spent sleeping. *Anat min* is time collecting anatomical images that were completed. *Scout min* is time collecting localizer data. *Excl motion min* is time collecting data excluded because of motion within a block, even though the block itself was usable. *Excl gaze min* is time collecting data excluded because the participant was not looking for an event within a block, even though the block itself was usable. *Excl block min* is the amount of time from blocks that were lost due to motion, not looking or because we quit. *Excl run min* is the amount of time for runs that cannot be included because all blocks were unusable. *Usable expts* is the number of usable experiments the participant contributed. *Run num* is the number of runs collected from this participant. The last five columns count the runs ended for documented reasons: *Finish ends*, the participant finished what we wanted to do in a run (although not necessarily enough for an experiment); *Fuss ends*, the participant became fussy or was moving excessively; *Sleep ends*, the participant fell asleep; *Tech ends*, technical issues (e.g., scanner stopped accidentally); *Unknown ends*: no reason documented. Participants who contributed zero usable scanning data are not listed. The last rows average the data within columns and also correlate these columns with age.

Ppt	Age	Sex	Cohort	Func min	Asleep min	Anat min	Scout min	Excl motion min	Excl gaze min	Excl block min	Excl run min	Usable expts	Run num	Finish ends	Fuss ends	Sleep ends	Tech ends	Unknown ends
s8687_1_1	3.7	F	2	2.7	0.0	1.8	0.2	0.4	0.0	5.8	0.0	0	1	1	0	0	0	0
s2687_1_1	4.0	M	2	4.8	0.0	4.9	0.2	1.0	0.0	2.9	0.0	1	1	1	0	0	0	0
s6607_1_1	4.0	M	2	9.5	0.0	3.1	0.7	1.0	0.0	5.2	0.0	2	2	1	1	0	0	0
s1687_1_1	4.5	M	2	0.0	0.0	3.1	0.7	0.0	0.0	0.0	4.1	0	2	0	2	0	0	0
s2607_1_1	4.5	F	2	1.6	0.0	3.1	0.2	0.7	0.0	2.1	0.9	0	2	0	2	0	0	0
s4607_1_1	4.6	F	2	4.5	0.0	3.1	0.2	0.7	0.0	1.4	0.0	0	1	0	1	0	0	0
s3687_1_1	4.7	F	2	2.3	0.0	4.9	0.5	0.7	0.0	5.6	1.2	0	3	1	1	1	0	0
s6687_1_1	5.0	F	2	8.6	0.0	3.1	0.2	2.0	0.0	4.3	0.0	2	2	2	0	0	0	0
s1607_1_1	5.1	M	2	2.6	0.0	3.1	0.2	1.3	0.1	1.9	2.3	1	2	1	1	0	0	0
s0687_1_1	5.3	F	2	4.5	0.0	3.1	0.5	0.5	0.0	2.1	0.0	1	1	0	1	0	0	0
s3687_1_2	5.7	F	2	3.0	0.0	3.1	0.5	0.5	0.0	1.8	4.3	1	2	0	1	0	1	0
s7687_1_1	5.8	M	2	0.0	4.9	4.9	0.5	0.0	0.0	0.0	0.0	0	2	2	0	0	0	0
s8687_1_2	5.8	F	2	4.8	0.0	9.4	0.5	0.3	0.0	0.0	2.6	2	3	3	0	0	0	0
s2687_1_2	6.0	M	2	11.7	0.0	8.1	0.2	1.2	0.1	1.4	0.0	3	4	3	0	1	0	0
s0307_1_1	6.3	M	1	4.7	9.6	6.3	0.7	0.4	0.8	0.8	0.2	0	4	0	1	1	0	2
s4607_1_2	6.7	F	2	7.7	0.0	3.1	0.5	0.8	0.6	0.7	0.0	2	3	2	1	0	0	0
s2187_1_1	7.3	F	1	2.3	0.0	9.4	0.2	0.2	3.1	1.1	0.0	1	2	2	0	0	0	0
s6687_1_2	7.3	F	2	5.8	0.0	3.1	0.2	2.2	0.4	2.0	0.0	2	2	1	0	0	1	0
s5687_1_1	7.4	M	2	1.4	0.0	3.1	1.2	1.0	0.0	2.9	4.9	0	5	0	4	0	1	0
s3607_1_1	7.5	F	2	8.0	0.0	3.1	0.7	1.0	0.0	1.5	0.0	1	2	1	0	0	1	0
s0607_1_1	7.6	M	2	3.7	0.0	3.1	0.7	0.1	0.0	0.7	5.3	0	3	0	2	0	1	0
s8187_1_1	8.5	F	1	5.4	0.0	0.0	0.5	0.6	0.0	0.0	1.7	0	6	0	0	0	0	6
s8607_1_1	8.5	F	2	12.3	0.0	4.9	0.2	0.9	0.6	2.1	0.0	3	2	2	0	0	0	0
s4107_1_1	8.8	M	1	2.6	2.1	6.3	0.7	1.2	0.9	2.4	4.2	1	4	2	0	2	0	0
s0687_1_2	9.0	F	2	4.5	0.0	6.3	0.5	0.1	0.3	2.1	1.7	2	3	1	1	1	0	0
s0307_1_2	9.1	M	1	13.5	0.0	6.3	0.5	1.0	0.6	1.4	0.0	3	3	2	0	1	0	0
s8187_1_2	9.4	F	1	10.5	0.0	3.1	0.7	1.3	3.3	0.9	0.9	2	7	1	3	0	2	1
s5687_1_2	10.4	M	2	3.3	0.0	0.0	0.7	1.5	0.0	1.5	0.0	1	1	0	1	0	0	0
s0187_1_1	10.5	M	1	0.8	0.0	6.3	0.7	0.0	0.0	2.3	2.4	0	2	1	1	0	0	0
s8187_1_3	10.8	F	1	2.7	0.0	3.1	0.5	1.0	0.0	4.0	3.9	1	3	1	0	0	2	0
s7307_1_1	11.2	F	1	2.1	0.0	0.0	0.7	0.9	0.0	1.4	0.0	0	1	0	1	0	0	0
s6187_2_1	11.3	M	1	9.1	0.0	3.1	0.5	0.4	1.8	0.9	0.0	2	3	1	0	0	0	2
s4107_1_2	12.2	M	1	0.7	0.0	3.1	0.7	0.1	0.0	0.7	2.3	0	2	1	1	0	0	0
s6187_2_2	12.2	M	1	1.9	0.0	0.0	0.5	1.4	0.4	1.8	0.0	0	2	0	2	0	0	0
s8187_1_4	13.8	F	1	7.2	6.0	9.4	0.7	1.3	0.0	4.0	0.0	2	4	2	1	1	0	0
s8187_1_5	15.3	F	1	4.4	0.0	6.3	0.7	1.3	0.0	6.0	0.0	0	3	1	1	0	1	0
s8187_1_6	16.8	F	1	5.2	0.0	3.1	0.9	0.8	0.0	2.7	1.5	1	4	1	2	0	1	0
s8187_1_7	18.2	F	1	17.0	0.0	6.3	0.5	1.4	0.7	0.9	0.0	2	4	3	1	0	0	0
s2307_1_1	19.9	M	1	18.0	2.1	6.3	0.2	0.2	0.2	1.9	0.0	3	5	2	0	1	2	0
s1187_1_1	20.7	F	1	13.1	0.0	6.3	0.2	0.8	0.8	0.0	0.0	3	3	2	1	0	0	0
s2307_1_2	21.7	M	1	20.2	0.0	6.3	0.2	0.1	0.4	0.7	0.0	3	4	1	0	3	0	0
s8187_1_8	23.1	F	1	13.5	0.0	6.3	0.2	0.6	0.1	2.2	0.0	3	3	3	0	0	0	0
s1187_1_2	23.2	F	1	10.8	0.0	6.3	0.7	2.5	0.8	0.0	2.6	2	5	2	3	0	0	0
s5187_1_2	26.3	F	1	5.9	0.0	3.1	2.1	0.8	0.0	0.4	3.4	2	6	4	0	0	2	0
s0307_2_1	32.6	M	1	5.1	0.0	3.1	0.2	0.0	0.0	0.0	1.5	2	3	2	1	0	0	0
Mean	10.7	NA	1	6.3	0.5	4.3	0.5	0.8	0.4	1.9	1.2	1.27	2.93	1.24	0.84	0.27	0.33	0.24
Under 12	7.1	NA	1	5.0	0.5	4.1	0.5	0.8	0.4	2.0	1.3	1.06	2.62	1.00	0.78	0.22	0.28	0.34
Over 12	19.7	NA	1	9.5	0.6	5.1	0.6	0.9	0.3	1.6	0.9	1.77	3.69	1.85	1.00	0.38	0.46	0.00
Cohort I	15.2	NA	1	7.7	0.9	4.8	0.6	0.8	0.6	1.6	1.1	1.43	3.61	1.48	0.83	0.39	0.43	0.48
Cohort II	6.0	NA	2	4.9	0.2	3.9	0.5	0.8	0.1	2.2	1.2	1.09	2.23	1.00	0.86	0.14	0.23	0.00
Corr with age	NA	NA	NA	0.45	-0.06	0.14	0.22	0.01	0.02	-0.28	-0.01	0.43	0.44	0.46	0.03	0.12	0.21	-0.08

Supplementary Table 3. Summary table for task-evoked activity in Cohort I. *Ppt* is the anonymized participant ID. *Run* indicates the run ID, with letters identifying pseudoruns. *Age* refers to the participant age in months. *TR num* refers to the number of TRs in the run (before blocks were removed). *Usable blocks* refers to the number of blocks in this run not excluded. *Total blocks* is the number of blocks from the run. *Block length* is the duration in seconds of the average block from this run. *Mean motion* is the translation plus rotational motion averaged across all TRs in the run in millimeters. *Mean excl TRs* is the number of TRs in this entire run that exceed the 3mm translational motion threshold. *Mean RMS* is the mean root mean squared error between functional volumes during the run. *Mean reliability* is the proportion of manually coded eye-gaze responses that matched the modal response, aggregated across coders. *Mean looking* is the proportion of time the participants were looking at the screen for all blocks in the run (regardless of exclusion). *Prop V1* is the proportion of significant voxels in V1. *Prop LOC* is the proportion of significant voxels in LOC. *Prop A1* is the proportion of significant voxels in A1. The last rows average the data within columns and also correlate these columns with age. Due to technical error, s8187_1_3 does not have recorded eye data but was monitored during the session for focus.

Ppt	Run	Age	TR num	Usable blocks	Total blocks	Block length	Mean motion	Mean excl TRs	Mean RMS	Mean reliability	Mean looking	Prop V1	Prop LOC	Prop A1
s0307_1_1	functional01	6.3	73	2	2	36.0	1.62	0.10	0.02	0.88	0.85	0.76	0.41	0.11
s0307_1_1	functional02	6.3	77	2	2	70.0	1.57	0.08	0.01	0.78	0.46	0.40	0.26	0.00
s4107_1_1	functional02	8.8	68	3	3	36.0	2.49	0.24	0.02	0.83	0.46	0.00	0.01	0.00
s4107_1_1	functional01	8.8	141	3	6	36.0	3.84	0.43	0.02	0.88	0.72	0.01	0.00	0.00
s0307_1_2	functional01	9.1	269	10	12	36.0	1.16	0.06	0.01	0.96	0.78	0.36	0.14	0.00
s0307_1_2	functional02	9.1	66	3	3	36.0	0.80	0.08	0.01	0.99	0.60	0.03	0.02	0.00
s8187_1_3	functional03	10.8	204	5	12	24.0	3.33	0.46	0.02	nan	nan	0.00	0.00	0.00
s6187_2_1	functional03	11.3	146	9	9	24.0	0.60	0.02	0.01	0.95	0.65	0.28	0.21	0.05
s6187_2_1	functional01	11.3	113	3	3	66.0	0.92	0.06	0.01	0.64	0.76	0.03	0.01	0.06
s6187_2_1	functional02	11.3	102	3	4	42.0	0.86	0.01	0.01	0.72	0.70	0.03	0.00	0.00
s6187_2_2	functional01	12.2	90	3	4	36.0	3.93	0.43	0.04	0.95	0.98	0.00	0.01	0.00
s8187_1_4	functional04b	13.8	70	2	3	36.0	3.08	0.29	0.03	0.88	1.00	0.00	0.00	0.00
s8187_1_4	functional01	13.8	156	5	7	36.0	1.22	0.04	0.01	0.86	0.63	0.00	0.01	0.00
s8187_1_4	functional04a	13.8	88	3	4	38.0	3.06	0.28	0.02	0.80	0.78	0.02	0.03	0.02
s8187_1_5	functional03	15.3	140	2	5	42.0	3.41	0.34	0.04	0.96	0.55	0.40	0.36	0.00
s8187_1_5	functional01	15.3	102	3	4	42.0	3.05	0.38	0.02	0.94	0.79	0.01	0.01	0.00
s2307_1_1	functional02	19.9	153	7	7	36.0	0.41	0.02	0.01	0.97	0.95	0.66	0.30	0.13
s2307_1_1	functional05a	19.9	144	4	4	65.0	0.28	0.01	0.00	0.87	0.94	0.42	0.41	0.18
s2307_1_1	functional01	19.9	112	3	6	30.7	0.79	0.03	0.01	0.85	0.59	0.04	0.01	0.00
s2307_1_1	functional04	19.9	118	3	4	52.0	0.20	0.00	0.00	0.87	0.97	0.52	0.58	0.44
s2307_1_1	functional03	19.9	65	3	3	36.0	0.20	0.00	0.00	0.99	0.98	0.16	0.15	0.01
s1187_1_1	functional01	20.7	266	12	12	36.0	0.73	0.05	0.00	0.98	0.88	0.34	0.11	0.00
s1187_1_1	functional03	20.7	98	6	6	24.0	1.67	0.10	0.01	0.95	0.70	0.00	0.00	0.00
s1187_1_1	functional02	20.7	69	3	3	36.0	0.67	0.03	0.01	0.99	0.98	0.40	0.32	0.23
s2307_1_2	functional01	21.7	266	11	12	36.2	0.31	0.03	0.00	0.97	0.85	0.38	0.36	0.00
s2307_1_2	functional03	21.7	216	6	6	65.0	0.12	0.00	0.00	0.84	0.91	0.44	0.42	0.06
s8187_1_8	functional02	23.1	263	9	12	36.0	2.10	0.20	0.01	0.97	0.78	0.02	0.00	0.00
s8187_1_8	functional03	23.1	65	3	3	36.0	0.81	0.00	0.01	0.99	0.94	0.01	0.06	0.01
s1187_1_2	functional05b	23.2	99	4	4	42.0	1.20	0.10	0.01	0.89	0.79	0.05	0.01	0.00
s1187_1_2	functional05a	23.2	95	4	4	40.0	1.95	0.23	0.01	0.92	0.83	0.06	0.06	0.09
s1187_1_2	functional03	23.2	95	3	3	58.7	1.68	0.14	0.01	0.93	0.84	0.01	0.08	0.00
s5187_1_2	functional06	26.3	55	2	3	31.3	18.00	0.27	0.06	0.97	0.90	0.00	0.01	0.04
Mean	NA	16.4	127.62	4.50	5.47	40.5	2.06	0.14	0.02	0.90	0.79	0.18	0.14	0.05
Under 12	NA	9.3	125.90	4.30	5.60	40.6	1.72	0.15	0.01	0.85	0.66	0.19	0.11	0.02
Over 12	NA	19.6	128.41	4.59	5.41	40.5	2.22	0.13	0.02	0.93	0.84	0.18	0.15	0.06
Corr with age	NA	NA	0.10	0.19	0.08	0.00	0.15	-0.25	-0.07	0.42	0.52	-0.05	0.10	0.20

Supplementary Table 4. Summary table for task-evoked activity in Cohort II. Refer to Supplementary Table 3 for a description of the header names.

Ppt	Run	Age	TR num.	Usable blocks	Total blocks	Block length	Mean motion	Mean excl TRs	Mean RMS	Mean reliability	Mean looking	Prop V1	Prop LOC	Prop A1
s8687_1_1	functional01	3.7	262	4	12	36.0	2.76	0.35	0.02	0.95	0.40	0.07	0.08	0.01
s6607_1_1	functional02d	4.0	53	2	2	45.0	0.35	0.00	0.01	0.89	1.00	0.20	0.07	0.19
s2687_1_1	functional01	4.0	258	8	12	36.0	3.06	0.31	0.01	0.91	0.86	0.00	0.00	0.00
s6607_1_1	functional02b	4.0	50	2	2	44.0	0.62	0.04	0.01	0.89	1.00	0.01	0.03	0.00
s6607_1_1	functional01	4.0	176	2	8	36.0	5.92	0.57	0.04	0.99	0.94	0.00	0.00	0.00
s2607_1_1	functional01	4.5	129	3	6	36.0	4.06	0.50	0.02	0.98	0.57	0.00	0.00	0.00
s3687_1_1	functional02	4.7	126	2	6	36.0	2.91	0.40	0.02	0.97	0.42	0.00	0.00	0.00
s6687_1_1	functional01a	5.0	259	11	12	36.2	2.19	0.23	0.01	0.94	0.80	0.00	0.00	0.00
s1607_1_1	functional01b	5.1	95	4	4	41.0	2.80	0.34	0.02	0.87	0.94	0.00	0.00	0.00
s1607_1_1	functional01a	5.1	75	2	4	30.0	6.78	0.63	0.04	0.88	0.77	0.00	0.01	0.02
s0687_1_1	functional01	5.3	211	7	10	36.0	1.67	0.18	0.01	0.96	0.69	0.34	0.11	0.00
s3687_1_2	functional02a	5.7	139	3	4	49.5	3.74	0.42	0.02	0.88	0.91	0.00	0.00	0.00
s8687_1_2	functional01	5.8	71	3	3	38.0	1.57	0.14	0.02	0.99	0.96	0.25	0.29	0.47
s2687_1_2	functional04	6.0	85	2	4	36.0	0.16	0.00	0.00	0.99	0.50	0.04	0.02	0.01
s2687_1_2	functional03	6.0	116	4	4	49.5	1.58	0.14	0.01	0.96	0.94	0.57	0.54	0.13
s2687_1_2	functional01	6.0	65	3	3	36.0	0.87	0.08	0.01	0.99	0.97	0.14	0.48	0.09
s4607_1_2	functional02a	6.7	102	4	4	44.0	0.89	0.02	0.01	0.95	0.91	0.01	0.12	0.03
s4607_1_2	functional01	6.7	65	3	3	36.0	1.50	0.12	0.01	0.94	0.68	0.00	0.00	0.00
s6687_1_2	functional02a	7.3	65	2	3	36.0	2.55	0.32	0.02	0.95	0.69	0.00	0.00	0.00
s3607_1_1	functional01	7.5	61	2	3	35.3	12.59	0.34	0.05	0.94	0.90	0.06	0.12	0.09
s3607_1_1	functional02a	7.5	216	10	10	36.0	1.48	0.10	0.01	0.99	0.90	0.54	0.28	0.00
s0607_1_1	functional01	7.6	131	5	6	36.0	0.28	0.02	0.01	0.96	0.81	0.28	0.09	0.04
s8607_1_1	functional02b	8.5	65	3	3	36.0	1.09	0.11	0.01	0.92	0.60	0.16	0.08	0.01
s8607_1_1	functional01	8.5	252	9	12	36.0	1.19	0.11	0.01	0.96	0.78	0.22	0.07	0.00
s0687_1_2	functional01	9.0	65	3	3	36.0	0.69	0.06	0.01	0.94	0.74	0.07	0.09	0.00
s5687_1_2	functional01b	10.4	108	3	6	31.0	4.69	0.48	0.02	0.91	0.88	0.24	0.13	0.25
Mean	NA	6.1	126.92	4.08	5.73	37.8	2.62	0.23	0.02	0.94	0.79	0.12	0.10	0.05
Correlation with age	NA	NA	-0.22	0.07	-0.16	-0.28	-0.01	-0.22	-0.05	0.08	0.04	0.32	0.20	0.15
