# Supplementary material for: Re-imagining fMRI for awake behaving infants
Source: Nat Commun. 2020 Sep 9;11:4523. doi: 10.1038/s41467-020-18286-y (PMC7481790; doi:10.1038/s41467-020-18286-y)
Supplement: Supplementary file 2 — Reporting Summary [file 41467_2020_18286_MOESM2_ESM.pdf]

nature research

Corresponding author(s): Nicholas B Turk-Browne

Last updated by author(s): Jul 8, 2020

Reporting Summary

Nature Research wishes to improve the reproducibility of the work that we publish. This form provides structure for consistency and transparency in reporting. For further information on Nature Research policies, see Authors & Referees and the Editorial Policy Checklist.

Statistics

For all statistical analyses, confirm that the following items are present in the figure legend, table legend, main text, or Methods section.

n/a	Confirmed	
☐	☒	The exact sample size (*n*) for each experimental group/condition, given as a discrete number and unit of measurement
☐	☒	A statement on whether measurements were taken from distinct samples or whether the same sample was measured repeatedly
☐	☒	The statistical test(s) used AND whether they are one- or two-sided<br>*Only common tests should be described solely by name; describe more complex techniques in the Methods section.*
☐	☒	A description of all covariates tested
☐	☒	A description of any assumptions or corrections, such as tests of normality and adjustment for multiple comparisons
☐	☒	A full description of the statistical parameters including central tendency (e.g. means) or other basic estimates (e.g. regression coefficient) AND variation (e.g. standard deviation) or associated estimates of uncertainty (e.g. confidence intervals)
☐	☒	For null hypothesis testing, the test statistic (e.g. *F*, *t*, *r*) with confidence intervals, effect sizes, degrees of freedom and *P* value noted<br>*Give P values as exact values whenever suitable.*
☒	☐	For Bayesian analysis, information on the choice of priors and Markov chain Monte Carlo settings
☐	☒	For hierarchical and complex designs, identification of the appropriate level for tests and full reporting of outcomes
☒	☐	Estimates of effect sizes (e.g. Cohen's *d*, Pearson's *r*), indicating how they were calculated

Our web collection on statistics for biologists contains articles on many of the points above.

Software and code

Policy information about availability of computer code

Data collection	Cognitive tasks were run with a custom experiment menu system based on Psychtoolbox (3.0) running in MATLAB (Mathworks). Video for eye-tracking was recorded with SMI iViewX (Cohort I) and custom Python (3.6) code in openCV (Cohort II). All code is available publicly at: github.com/experiment_menu
Data analysis	Infant fMRI data were analyzed with a custom pipeline that wrapped tools from FSL (5.0.9) and AFNI (June 2016) using MATLAB (Mathworks), Python (3.6), and bash scripts. All code is available publicly at: github.com/infant_neuropipe

For manuscripts utilizing custom algorithms or software that are central to the research but not yet described in published literature, software must be made available to editors/reviewers. We strongly encourage code deposition in a community repository (e.g. GitHub). See the Nature Research guidelines for submitting code & software for further information.

Data

Policy information about availability of data

All manuscripts must include a data availability statement. This statement should provide the following information, where applicable:

- Accession codes, unique identifiers, or web links for publicly available datasets
- A list of figures that have associated raw data
- A description of any restrictions on data availability

Data will be made publicly available on Dryad upon publication at: https://doi.org/10.5061/dryad.8ghtt76k3. The two included datasets (Cohorts I and II) are sufficient to recreate: Figures 2 (infants), 3, 4, 5, 6; Supplementary Figures 3 (infants), 4 (infants), 5, 6; and Supplementary Tables 2, 3, 4. Code is provided to generate these images from the shared data. The source data for all figures is provided in the Source_data.xls file.

Field-specific reporting

Please select the one below that is the best fit for your research. If you are not sure, read the appropriate sections before making your selection.

☐ Life sciences ☒ Behavioural & social sciences ☐ Ecological, evolutionary & environmental sciences

For a reference copy of the document with all sections, see nature.com/documents/nr-reporting-summary-flat.pdf

Behavioural & social sciences study design

All studies must disclose on these points even when the disclosure is negative.

Study description	This was a quantitative experimental study using a cross-sectional design of human infants
Research sample	In Cohort I, 11 unique infants (5 females) aged 6 to 33 months were scanned across 23 sessions (1–8 sessions per participant), sampled from families in the Princeton and surrounding Mercer County, NJ areas. Not included in this total were 5 sessions without fMRI data because the infant would not lie down (4 additional unique infants, 1 infant included above who contributed a usable session on another occasion). In Cohort II, 15 unique infants (8 females) aged 4 to 10 months were scanned across 22 sessions (1–2 sessions per participant), sampled from families giving birth at the Yale-New Haven Hospital in New Haven, CT. One other session was excluded because the infant would not lie down (1 additional unique infant). All of these details are reported in the paper.
Sampling strategy	Two cohorts were collected sequentially at different locations using a convenience sample of interested families. We recruited as many participants as possible for both cohorts, given the difficulty of recruitment, unpredictability of scan success, and dearth of this kind of data. Each experiment we ran in these cohorts had planned sample sizes based on piloting and prior adult studies. No prior infant fMRI studies were available to estimate statistical power. However, based on adult fMRI studies, this sample size would be sufficient to elicit task-evoked activity. We also successfully replicated key findings across cohorts, choosing preprocessing and analysis parameters in Cohort I and applying them in Cohort II.
Data collection	fMRI data were collected on a Siemens Prisma 3T MRI machine and eye-tracking data were collected using an MRC camera inside the scanner bore. At least one parent and one expert experimenter was present in the scanner room at all times during data collection, with at least two additional experiments in the control room. The parents were not informed of the experimental condition (and obviously not the infants). The experimenters were not blinded but could not be seen by the infant during the scan.
Timing	Cohort I was collected between February 2016 and June 2017. Cohort II was collected between August 2018 and December 2018.
Data exclusions	All attempts at scanning infants between 3 and 36 months are reported in the paper. For the amount of usable data per scan (Figure 1), we do not plot participants who did not go into the scanner. For the SFNR/SNR analyses, we use all functional runs regardless of length. For the task-based univariate analyses, we do not analyze runs that did not have at least two usable task blocks of the same experiment. This planned exclusion reflects our threshold of two blocks to form a GLM, chosen to provide a minimum amount of averaging across blocks while being as liberal as possible in order to include as much of these precious data as possible.
Non-participation	In Cohort I, of the families who attended an orientation session, 15 children participated in at least one scan and 3 were either not invited back for a scan or were invited but declined (reasons unknown). In Cohort II, of the families who attended an orientation session, 16 children participated in at least one scan, 1 was not invited back for a scan, and 2 were invited but could not be scheduled.
Randomization	All experiments employed within-subjects designs and thus there was no randomization to groups across participants. However, we did randomly counterbalance stimulus sets and condition orders across participants when applicable. Which tasks infants performed in each given session depended on: (1) what they had completed in previous sessions (if any); (2) whether the task was age-appropriate for the infant; (3) whether the infant would tolerate the stimuli without fussing out. The only covariates used in these analyses were age and preprocessing parameters, which were not randomly assigned.

Reporting for specific materials, systems and methods

We require information from authors about some types of materials, experimental systems and methods used in many studies. Here, indicate whether each material, system or method listed is relevant to your study. If you are not sure if a list item applies to your research, read the appropriate section before selecting a response.

Materials & experimental systems

n/a	Involved in the study	
☒	☐	Antibodies
☒	☐	Eukaryotic cell lines
☒	☐	Palaeontology
☒	☐	Animals and other organisms
☐	☒	Human research participants
☒	☐	Clinical data

Methods

n/a	Involved in the study	
☒	☐	ChIP-seq
☒	☐	Flow cytometry
☐	☒	MRI-based neuroimaging

Human research participants

Policy information about studies involving human research participants

Population characteristics	See above.
Recruitment	Participants in Cohort I were recruited through flyers, outreach events, and word of mouth. Participants in Cohort II were recruited via maternity ward visits to the Yale-New Haven Hospital soon after birth. There was likely a self-selection bias based on the parents being willing to participant; however, we did not collect data on the parents.
Ethics oversight	The Princeton University and Yale University Institutional Review Boards reviewed and approved the research protocols for Cohort I and Cohort II, respectively. This is reported in the paper.

Note that full information on the approval of the study protocol must also be provided in the manuscript.

Magnetic resonance imaging

Experimental design

Design type	Most of the data were collected during tasks with an ON-OFF or alternating A-B block design.
Design specifications	Participants completed a variety of task blocks spanning several experiments. Of interest here, we analyzed blocks lasting 24–80s, with 2–12 usable blocks within individual runs. Blocks were separated by at least 6s of rest.
Behavioral performance measures	We recorded eye-tracking data from the infants. Only blocks where the infant was looking at the screen for more than 50% of the block were retained.

Acquisition

Imaging type(s)	Functional and structural scans were acquired
Field strength	3T
Sequence & imaging parameters	For anatomical scans, we used a T1-weighted PETRA sequence in all participants TR(1)=3.32ms, TR(2)=2250ms, TE=0.07ms, flip angle=6 degrees, matrix=320x320, slices=320, resolution=0.94mm isotropic, radial lines=30,000). In two young infants, we additionally piloted a T2-weighted SPACE sequence (TR=3200ms, TE=563ms, flip angle=120 degrees, matrix=192x192, slices=176, resolution=1mm isotropic). For functional scans, we used a T2*-weighted gradient-echo EPI sequence in all participants (TR=2000ms, TE=28ms, flip angle=71 degrees, matrix=64x64, slices=36, resolution=3mm isotropic, interleaved slice acquisition) covering the whole brain.
Area of acquisition	Whole-brain acquisition

Diffusion MRI ☐ Used ☒ Not used

Preprocessing

Preprocessing software	We developed and released a custom pipeline for preprocessing and analysis of infant fMRI data. This pipeline incorporates AFNI for skull-stripping and FSL for linear de-trending, motion correction (based on the centroid volume), slice-time correction, and spatial smoothing.
Normalization	Functional data were z-scored within voxel over time for each run. Motion corrected volumes were aligned to the infant's anatomical scan, which was in turn aligned to an age-appropriate infant atlas and adult MNI space.
Normalization template	Age-appropriate infant atlas (Fonov, 2009) and adult MNI space (MNI152)
Noise and artifact removal	Motion parameters accounting for 6 degrees of freedom were regressed out.
Volume censoring	Time points that exceeded 3-mm of translational motion were excised, with values interpolated from adjacent time points before linear de-trending and then regressed out in the GLM.

Statistical modeling & inference

Model type and settings	Mass univariate GLMs were performed at the first level (for each run or pseudo-run).
Effect(s) tested	SFNR was computed for each run by dividing the mean BOLD activity in each voxel by the standard deviation of the detrended voxel activity. SFNR was compared along the anterior-posterior axis by randomly sampling and then averaging voxels in every coronal brain slice. SNR was calculated at the centroid TR by dividing the mean of 1000 brain voxels by the standard deviation of 500 non-brain voxels across the anterior-posterior axis. Visual evoked BOLD activity was quantified separately for each run as the proportion of voxels in each anatomical ROI with a significant ($p<.05$) GLM contrast of task vs. rest. Exploratory whole-brain analyses were conducted by performing a voxelwise t-test across all usable runs for the task vs. rest contrast. Preprocessing decisions were evaluated by varying the following parameters

and comparing the proportion of voxels with significant visual evoked BOLD activity in each usable run and ROI: exclusion threshold for translational motion, number of time-points excluded after motion, extent of spatial smoothing, ICA-motion correlation threshold for de-noising, voxelwise despiking, and inclusion of temporal derivatives. We repeated all of these analyses at the level of sessions rather than runs, after concatenating all runs and usable blocks within each session.

Specify type of analysis: ☐ Whole brain ☐ ROI-based ☒ Both

Anatomical location(s): V1, LOC and A1 were defined from the probabilistic Harvard-Oxford atlas, with probability threshold 0.

Statistic type for inference (See Eklund et al. 2016): For whole-brain analyses, p-values from voxelwise t-test of task vs. rest.

Correction: None, whole-brain analyses were secondary and exploratory

Models & analysis

n/a	Involved in the study
☒	☐ Functional and/or effective connectivity
☒	☐ Graph analysis
☒	☐ Multivariate modeling or predictive analysis
