# Supplementary material for: Re-imagining fMRI for awake behaving infants
Source: Nat Commun. 2020 Sep 9;11:4523. doi: 10.1038/s41467-020-18286-y (PMC7481790; doi:10.1038/s41467-020-18286-y)
Supplement: Supplementary file 3 — Description of Additional Supplementary Files [file 41467_2020_18286_MOESM3_ESM.pdf]

Description of Additional Supplementary Files

File Name: Supplementary Movie 1
Description: Video showing all time-points of three functional runs acquired from infants. The first is an example of a run with low motion, the second moderate motion, and the third severe motion. Coded names correspond to those used in the data release.
